# Global identification and characterization of tRNA-derived RNA fragment landscapes across human cancers

**DOI:** 10.1093/narcan/zcaa031

**Published:** 2020-10-19

**Authors:** Xiwei Sun, Juze Yang, Mengqian Yu, Dongxia Yao, Liyuan Zhou, Xufan Li, Qiongzi Qiu, Weiqiang Lin, Bingjian Lu, Enguo Chen, Ping Wang, Wantao Chen, Sifeng Tao, Haiming Xu, Anna Williams, Yong Liu, Xiaoqing Pan, Allen W Cowley, Weiguo Lu, Mingyu Liang, Pengyuan Liu, Yan Lu

**Affiliations:** Sir Run Run Shaw Hospital and Institute of Translational Medicine, School of Medicine, Zhejiang University, Hangzhou, Zhejiang 310016, China; Institute for Advanced Research, Wenzhou Medical University, Wenzhou, Zhejiang 325000, China; Sir Run Run Shaw Hospital and Institute of Translational Medicine, School of Medicine, Zhejiang University, Hangzhou, Zhejiang 310016, China; Center for Uterine Cancer Diagnosis & Therapy Research of Zhejiang Province, Women’s Reproductive Health Key Laboratory of Zhejiang Province, Department of Gynecologic Oncology, Women’s Hospital and Institute of Translational Medicine, School of Medicine, Zhejiang University, Hangzhou, Zhejiang 310006, China; Center for Uterine Cancer Diagnosis & Therapy Research of Zhejiang Province, Women’s Reproductive Health Key Laboratory of Zhejiang Province, Department of Gynecologic Oncology, Women’s Hospital and Institute of Translational Medicine, School of Medicine, Zhejiang University, Hangzhou, Zhejiang 310006, China; Sir Run Run Shaw Hospital and Institute of Translational Medicine, School of Medicine, Zhejiang University, Hangzhou, Zhejiang 310016, China; Sir Run Run Shaw Hospital and Institute of Translational Medicine, School of Medicine, Zhejiang University, Hangzhou, Zhejiang 310016, China; Center for Uterine Cancer Diagnosis & Therapy Research of Zhejiang Province, Women’s Reproductive Health Key Laboratory of Zhejiang Province, Department of Gynecologic Oncology, Women’s Hospital and Institute of Translational Medicine, School of Medicine, Zhejiang University, Hangzhou, Zhejiang 310006, China; The First Affiliated Hospital and Institute of Translational Medicine, School of Medicine, Zhejiang University, Hangzhou, Zhejiang 310003, China; Center for Uterine Cancer Diagnosis & Therapy Research of Zhejiang Province, Women’s Reproductive Health Key Laboratory of Zhejiang Province, Department of Gynecologic Oncology, Women’s Hospital and Institute of Translational Medicine, School of Medicine, Zhejiang University, Hangzhou, Zhejiang 310006, China; Sir Run Run Shaw Hospital and Institute of Translational Medicine, School of Medicine, Zhejiang University, Hangzhou, Zhejiang 310016, China; The First Affiliated Hospital and Institute of Translational Medicine, School of Medicine, Zhejiang University, Hangzhou, Zhejiang 310003, China; Shanghai Ninth People’s Hospital, School of Medicine, Shanghai Jiaotong University, Shanghai 200011, China; The Second Affiliated Hospital, School of Medicine, Zhejiang University, Hangzhou, Zhejiang 310003, China; Institute of Bioinformatics, Zhejiang University, Hangzhou, Zhejiang 310058, China; Center of Systems Molecular Medicine, Department of Physiology, Medical College of Wisconsin, Milwaukee, WI 53226, USA; Center of Systems Molecular Medicine, Department of Physiology, Medical College of Wisconsin, Milwaukee, WI 53226, USA; Center of Systems Molecular Medicine, Department of Physiology, Medical College of Wisconsin, Milwaukee, WI 53226, USA; Department of Mathematics, Shanghai Normal University, Xuhui, Shanghai 200234, China; Center of Systems Molecular Medicine, Department of Physiology, Medical College of Wisconsin, Milwaukee, WI 53226, USA; Center for Uterine Cancer Diagnosis & Therapy Research of Zhejiang Province, Women’s Reproductive Health Key Laboratory of Zhejiang Province, Department of Gynecologic Oncology, Women’s Hospital and Institute of Translational Medicine, School of Medicine, Zhejiang University, Hangzhou, Zhejiang 310006, China; Cancer Center, Zhejiang University, Hangzhou, Zhejiang 310029, China; Center of Systems Molecular Medicine, Department of Physiology, Medical College of Wisconsin, Milwaukee, WI 53226, USA; Sir Run Run Shaw Hospital and Institute of Translational Medicine, School of Medicine, Zhejiang University, Hangzhou, Zhejiang 310016, China; Center of Systems Molecular Medicine, Department of Physiology, Medical College of Wisconsin, Milwaukee, WI 53226, USA; Cancer Center, Zhejiang University, Hangzhou, Zhejiang 310029, China; Center for Uterine Cancer Diagnosis & Therapy Research of Zhejiang Province, Women’s Reproductive Health Key Laboratory of Zhejiang Province, Department of Gynecologic Oncology, Women’s Hospital and Institute of Translational Medicine, School of Medicine, Zhejiang University, Hangzhou, Zhejiang 310006, China; Cancer Center, Zhejiang University, Hangzhou, Zhejiang 310029, China

## Abstract

Transfer RNA-derived RNA fragments (tRFs) are a class of small non-coding RNAs that are abundant in many organisms, but their role in cancer has not been fully explored. Here, we report a functional genomic landscape of tRFs in 8118 specimens across 15 cancer types from The Cancer Genome Atlas. These tRFs exhibited characteristics of widespread expression, high sequence conservation, cytoplasmic localization, specific patterns of tRNA cleavage and conserved cleavage in tissues. A cross-tumor analysis revealed significant commonality among tRF expression subtypes from distinct tissues of origins, characterized by upregulation of a group of tRFs with similar size and activation of cancer-associated signaling. One of the largest superclusters was composed of 22 nt 3′-tRFs upregulated in 13 cancer types, all of which share the activation of Ras/MAPK, RTK and TSC/mTOR signaling. tRF-based subgrouping provided clinically relevant stratifications and significantly improved outcome prediction by incorporating clinical variables. Additionally, we discovered 11 cancer driver tRFs using an effective approach for accurately exploring cross-tumor and platform trends. As a proof of concept, we performed comprehensive functional assays on a non-microRNA driver tRF, 5′-IleAAT-8-1-L20, and validated its oncogenic roles in lung cancer *in vitro* and *in vivo*. Our study also provides a valuable tRF resource for identifying diagnostic and prognostic biomarkers, developing cancer therapy and studying cancer pathogenesis.

## INTRODUCTION

Transfer RNA-derived RNA fragments (tRFs) are a class of small non-coding RNAs (ncRNAs) that are abundant and conserved across most organisms (1–8). These fragments are generated from the cleavage in the stem and loop structure of mature tRNAs or 3′ trailer sequences of their precursor transcripts under either stressed or unstressed conditions. Generally, at least five types of tRFs have been defined based on their cleavage sites in tRNAs: 5′- and 3′-halves (>30 nt), 5′- and 3′-tRFs (15–30 nt) and 3′U-tRFs (also named as tsRNAs) (8–10). Recently, 5′-tRFs were reported to increase in abundance during sperm maturation and act as regulators of offspring metabolism (11–13). This can be achieved through suppression of endogenous retroelement-regulated genes, although the mechanism is unknown. Moreover, a recent study showed that 3′-tRFs can silence mobility of long terminal repeat retrotransposons in mouse cells through inhibition of reverse transcription and RNAi silencing machinery, respectively (14). These results indicate that endogenous tRNA-related fragments are biologically functional molecules rather than random degradation products of tRNAs. Biogenesis and discovery of tRFs as well as their potential roles in various biological processes have been further described in recent reviews (15–17).

So far, the roles of tRNA fragments have only been explored in tumor development and progression in a handful of studies. Goodarzi *et al.* found that tRFs from tRNA^Glu^, tRNA^Asp^, tRNA^Gly^ and tRNA^Tyr^ can competitively bind to oncogenic protein, YBX1, with pro-oncogenic transcripts, resulting in inhibition of tumor metastasis in breast cancer cells (18). Honda *et al.* reported a group of 5′-halves whose expression levels were sex hormone dependent and were involved in cell proliferation in breast and prostate cancer (19). Additionally, dysregulation of 3′U-tRFs was discovered in multiple malignancies (20,21). Recently, Kim *et al.* showed that inhibition of a specific tRNA fragment, LeuCAG3′tsRNA, could induce apoptosis of hepatocellular cells *in vivo* and *in vitro* (22).

However, the biological and clinical significance of 5′- and 3′-tRFs in solid tumors is not yet clear. We suspect that they can also have oncogenic or tumor suppressor roles in cancer development and progression similar to the families of tRFs described above and well-characterized microRNAs (miRNAs). Evidence for this is urgently required. Furthermore, a comprehensive cross-tumor landscape of endogenous tRNA-derived fragments could greatly expand our current knowledge on the biogenesis, characteristics and function of these tRFs in cancer. This information could enable the discovery of potential robust biomarkers and therapeutic targets, which have not been previously identified.

In this study, we have systematically analyzed 5′- and 3′-tRF profiles using 8118 small RNA sequencing (smRNA-seq) datasets from 15 common cancer types with large sample sizes in The Cancer Genome Atlas (TCGA) in an effort to address the above questions. As a proof of concept, we performed comprehensive functional assays on a cancer-associated 5′-tRF, 5′-IleAAT-8-1-L20, and validated its oncogenic role in lung cancer *in vitro* and *in vivo*.

## MATERIALS AND METHODS

### Characterization of the expression profiles of tRFs

tRF annotation for mapping and quantifying tRFs was created as described previously (23). miRNA-seq BAM files of 7512 human tumor samples across 15 common cancer types and 606 corresponding normal tissue specimens (if available) were downloaded from the Genomic Data Commons (https://portal.gdc.cancer.gov/). These BAM files contained both mapped and unmapped sequence reads. First, mapped reads in these BAM files were remapped to sequence sets of our CCA-tRNA and pre-tRNA annotation using the Burrows–Wheeler transform algorithm (24), allowing for no mismatches per read. Then, these remapped reads were used to count the number of reads belonging to each of the candidate tRFs. Finally, the expression of tRFs was quantified as reads per million mapped reads (RPM), which has been commonly used in previous miRNA studies (25).

### Subcellular localization of tRFs

Short RNA-seq data of subcellular compartments (nucleus and cytoplasm) of A549 cell line in the ENCODE project were obtained from Gene Expression Omnibus (GSE24565) and used to quantify the expression of miRNAs, small nuclear RNAs (snRNAs), small nucleolar RNAs (snoRNAs), small cytoplasmic RNAs (scRNAs) and Piwi-interacting RNAs, as detailed in the Supplementary Methods.

### Jensen–Shannon divergence cleavage score

The Jensen–Shannon (JS) divergence is used to compare two discrete probability distributions, and is calculated as follows:

(1) $$\begin{equation*} { {{\bf JS}}}\left( {{\boldsymbol e}^{\boldsymbol 1}},{{\boldsymbol e}^{\boldsymbol 2}} \right) = {\boldsymbol H}\left( {\frac{{{{\boldsymbol e}^1} + {{\boldsymbol e}^2}}}{ 2}} \right) - \frac{{{\boldsymbol H}\left( {{{\boldsymbol e}^1}} \right) + {\boldsymbol H}\left( {{{\boldsymbol e}^2}} \right)}}{ 2}, \end{equation*}$$

where ***H*** is the Shannon entropy of the distribution:

$$\begin{equation*}{{\boldsymbol e}^{\boldsymbol 1}}{\rm{\;and}}\;{{\boldsymbol e}^{\boldsymbol 2}} = \left( {{e_1},{e_2}, \ldots ,{e_n}} \right),\hbox{ where }0 \le {e_i} \le 1\hbox{ and }\mathop \sum \limits_{i = 1}^n {e_i} = 1,\end{equation*}$$

(2) $$\begin{equation*}{\boldsymbol H}\left( {{{\boldsymbol e}^{\boldsymbol 1}}} \right)\;{\rm{and}}\;{\boldsymbol H}\left( {{{\boldsymbol e}^{\boldsymbol 2}}} \right) = - \mathop \sum \limits_{i = 1}^n {e_i}\log \left( {{e_i}} \right).\end{equation*}$$

To quantify the distribution pattern of cleavage among different cancer types (or tissues) and tRNA genes, we defined a JS cleavage score that was modified from methods developed by Cabili *et al.* (26), as detailed in the Supplementary Methods.

### RPPA protein, miRNA-seq and mRNA-seq data

Reverse-phase protein lysate array (RPPA), miRNA and mRNA expression profile data of the studied 15 common cancer types in TCGA were downloaded from ICGC Data Portal (https://dcc.icgc.org) (Supplementary Table S1). Processing and filtering of these data are detailed in the Supplementary Methods.

### Functional assays

*In vitro* and *in vivo* assays for evaluating the functional role of 5′-IleAAT-8-1-L20 in lung cancer are described in the Supplementary Methods.

## RESULTS

### Identification, quantification and characterization of tRFs across 15 cancer types

**Figure 1.**
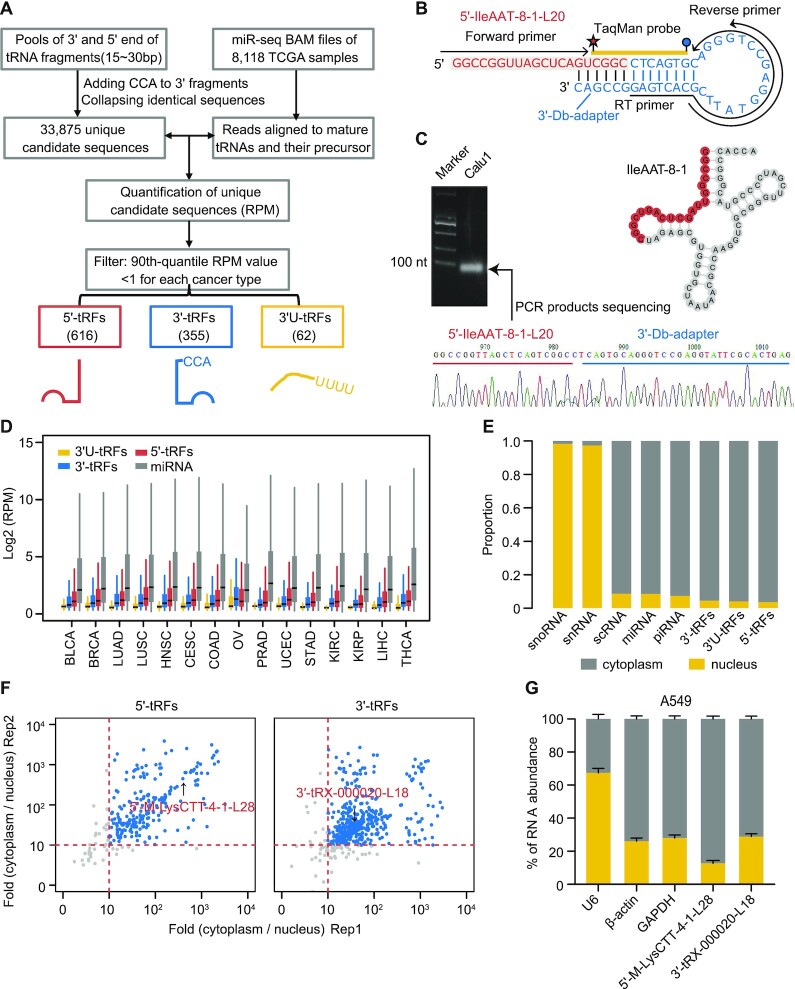
Identification and quantification of endogenous tRFs across 15 cancer types. (**A**) Schematic illustration of workflow to detect and quantify tRFs. (**B, C**) Dumbbell polymerase chain reaction (Db-PCR) was used to validate a 20 nt fragment (5′-IleAAT-8-1-L20) from 5′ end of tRNA^IleAAT-8-1^ that was identified using our computational pipeline. The PCR products were then sequenced. Integrated sequence and structure of Db-PCR adapter (light blue), fragment (dark yellow) and primer (red) are shown in panel (B). (**D**) Distribution of tRFs and miRNA expression across 15 cancer types. (**E**) Comparison of the relative abundance of nucleus versus cytoplasm in ENCODE A549 cell lines across different small ncRNA classes. The total reads of each small ncRNA class were normalized by RPM and then their relative abundance between the nuclear and cytoplasmic fractions was calculated. (**F**) Scatter plot of cytoplasmic over nuclear enrichment for 5′-tRFs (left panel) and 3′-tRFs (right panel) between two replicates in ENCODE A549 cells. (**G**) Db-PCR analyses of 5′-M-LysCTT-4-1-L28 and 3′-tRX-000020-L18 in the cytoplasm and nucleus of A549 cell lines. The two tRFs showed moderate expression abundance in A549 cells and were randomly selected for independent Db-PCR validation. β-Actin and GAPDH are used as the cytoplasmic control and U6 as the nuclear control. The error bars indicate standard deviation (SD) of three independent experiments. See also Supplementary Figure S1.

Identification of tRFs from smRNA-seq data presents two main challenges: (i) lack of evidence-based tRF annotation and (ii) multiple potential mapping loci in the human genome for most tRFs. Standard analysis procedures for miRNAs are unable to effectively mine these fragments (27). Accordingly, we developed a computational workflow for *de novo* tRF mining from sequencing libraries of small RNAs (Figure 1A). The expression level of tRFs was quantified using RPM normalization strategy, the commonly used method for miRNA analysis (25). To ensure robust detection, candidates whose 90th quantile RPM value was <1 for each cancer type were filtered, and the subsequent analysis was then focused on those remaining. In total, 616 5′-tRFs, 355 3′-tRFs and 62 3′U-tRFs shared by at least one cancer type were revealed, of which 211 (34.25%), 94 (26.48%) and 4 (6.45%) were commonly identified for all 15 cancer types, respectively (Supplementary Table S1).

It has been reported that tRNAs (and tRFs) are heavily modified and these modifications may lead to biased quantification of tRFs using smRNA-seq libraries. To evaluate the impact of tRNA modification on the quantification of tRFs, we analyzed tRFs in cell lines generated from public database using smRNA-seq and AlkB-facilitated sequencing (ARM-seq) methods, respectively (28). The sequencing data were downloaded from the Sequence Read Archive (SRP056032). In ARM-seq, RNA was treated with a dealkylating enzyme, *Escherichia coli* AlkB, before reverse transcription in library preparation. Two human B lymphocyte-derived cell lines (GM05372 and GM12878) were used in this study. Sequencing libraries of AlkB-treated and untreated RNA were prepared using a NEBNext Small RNA Library Prep Kit for an Illumina sequencer. Read mapping and quantification of tRFs in these datasets were analyzed by our tRF computational pipeline. Although smRNA-seq (i.e. sequencing from untreated samples) missed some tRFs compared with ARM-seq, the two methods showed high concordance in the quantification of tRFs, especially for 3'-tRF (Pearson’s *r* > 0.9, *P*-value <0.001) (Supplementary Figure S1A and B). These results were expected given the fact that the smRNA-seq method used by the TCGA involved ligating both adapters before the reverse transcription step. In fact, if tRNA chemical modification results in pausing of the reverse transcription, then the corresponding molecule was not amplified and finally would not emerge in the sequencing reads (29). In other words, the detected tRFs in TCGA datasets were accurately quantified.

In addition, because tRFs often have multiple terminal heterogeneity, traditional methods such as northern blot analysis or TaqMan reverse transcriptase PCR (RT-PCR) do not effectively quantify their abundance. To assess the robustness of our detected fragments, a quantitative Db-PCR analysis was performed in tumor specimens of a 20 nt 5′-tRF originating from tRNA^IleAAT-8-1^, termed 5′-IleAAT-8-1-L20 hereafter (Figure 1B; see Supplementary Methods). Db-PCR is a TaqMan qRT-PCR-based method and can distinctively quantify 5′ and 3′ end variants of RNA fragments at the single-base resolution (30). Db-PCR and northern blot methods were compared for measuring the abundance of 5′-IleAAT-8-1-L20 in lung cancer cell lines, A549 and Calu1. The two methods produced highly concordant measurements of tRF expression (Supplementary Figure S1C). The sequence of 5′-IleAAT-8-1-L20 was further verified by Sanger sequencing of Db-PCR products, confirming the credibility of Db-PCR (Figure 1C). Db-PCR was thus used for measuring the abundance of tRFs in subsequent validation experiments.

The distribution of tRF expression relative to miRNAs in multiple cancer types is shown in Figure 1D (see Supplementary Figure S1D for corresponding normal tissues). Across cancer types and normal tissues, it was observed that the abundance of some tRFs was of the same order of magnitude as certain miRNAs. Compared with other tissues, the global expression levels of tRFs were relatively elevated in kidney, liver and thyroid tissue, suggesting the tissue-specific expression of these fragments (Supplementary Figure S1D). As with miRNAs, the tRF expression level was skewed with an extremely wide distribution. The top five fragments accounted for almost half of the total abundance for each tumor or normal sample (Supplementary Figure S1F). The 5′- and 3′-tRFs in contrast to 3′U-tRFs were highly evolutionarily conserved similarly to exons and miRNAs (Supplementary Figure S1E), indicating that they were under highly selective pressure and thus of functional importance. This is not unexpected considering that tRNAs are strongly conserved and that 5′- and 3′-tRFs have been reported in yeast, bacteria, plants and mammals (31–34).

The subcellular localization of tRFs was then determined using short RNA-seq data of two main subcellular compartments (nucleus and cytoplasm) from the A549 cell line of the ENCODE project. The annotated small RNAs presented distinct enrichment in the nucleus and cytoplasm (Figure 1E; Supplementary Figure S1G and H). As is known, snRNAs and snoRNAs were found largely in the nucleus, whereas scRNA and miRNA were largely in the cytoplasm (35). Of note, these expected observations (as positive controls) suggest that the protocols for generating nuclear–cytoplasmic fractionation and smRNA-seq libraries were efficient, which in turn ensured the robustness of our results. Specifically, tRFs including 5′-tRFs and 3′-tRFs were found to be highly enriched in the cytoplasm (Figure 1F). Independent Db-PCR validation of randomly selected 5′-tRFs and 3′-tRFs with moderate expression abundance in A549 cells (shown in Figure 1F) further confirmed that these fragments are predominantly cytosolic (Figure 1G). Together, the results suggest that cytosolic distribution of these fragments may be related to their underlying functions.

### tRFs result from specific cleavage of tRNAs in cancer

**Figure 2.**
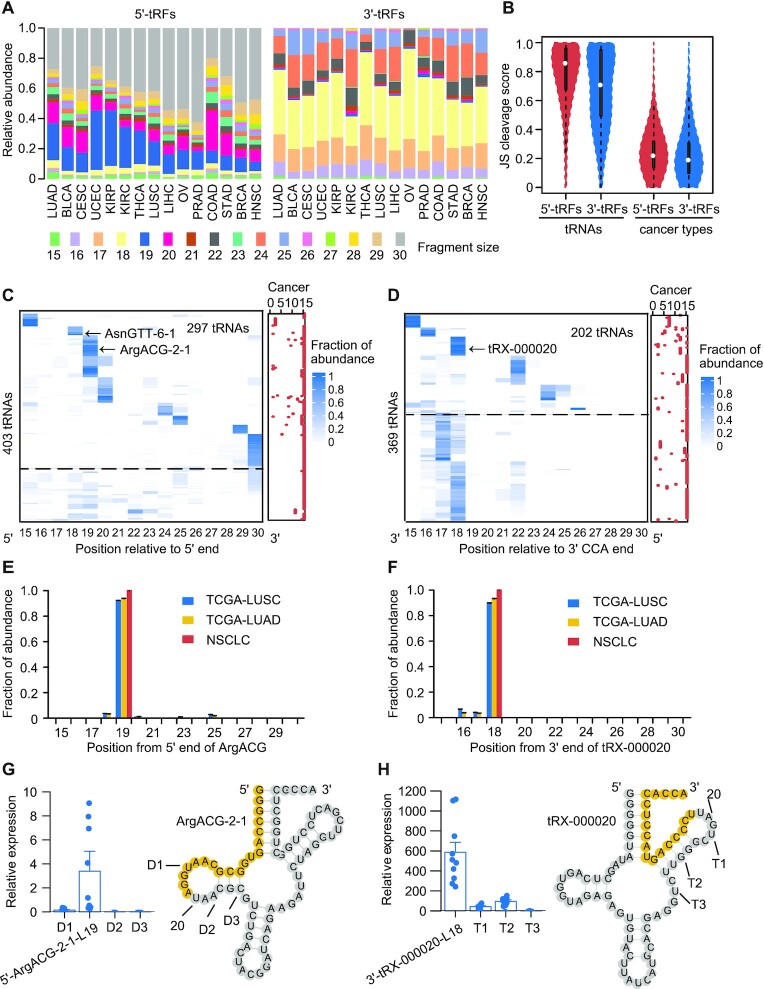
tRFs result from specific cleavage of tRNAs in cancer. (**A**) Relative abundance of different fragment sizes (15–30 nt) of 5′-tRFs (left panel) and 3′-tRFs (right panel) across cancer types. Shown are the average relative expression values for each fragment size across samples. (**B**) Distribution of JS cleavage score calculated across different tRNAs or cancer types. The JS cleavage score used Jensen–Shannon divergence (ranging from 0 to 1) as metric and JS = 0 represented the same cleavage pattern among tRNAs or cancer types. Cleavage profiles of 5′ (**C**) and 3′ (**D**) ends of tRNAs that could generate 5′-tRFs and 3′-tRFs, respectively. Color intensity signifies relative ratio between reads mapped to a given position of tRNAs and total reads mapped to 5′ or 3′ end of tRNAs. Red dots in the right panel represent the number of cancer types harboring a similar cleavage pattern. Above the dashed line indicates highly specific cleavage of 5′ and 3′ end tRNAs. Examples of two tRNAs, tRNA^ArgACG-2-1^ [**E**, also shown in panel (C)] and tRNA^tRX-000020^ [**F**, also shown in panel (D)], that were specifically processed in their 5′ and 3′ ends, respectively, inferred from both TCGA and our lung cancer dataset. TCGA-LUSC and TCGA-LUAD are lung squamous cell carcinoma (LUSC) and lung adenocarcinoma (LUAD) datasets from TCGA; NSCLC is our non-small cell lung cancer dataset. Db-PCR analyses of fragments derived from the major and other three minor cleavage positions of the two above-described tRNAs, tRNA^ArgACG-2-1^ (**G**) and tRNA^tRX-000020^ (**H**), respectively. D1, D2 and D3 represent 5′ end fragments; T1, T2 and T3 represent 3′ end fragments; the error bars indicate SD across samples (*n* = 10). See also Supplementary Figure S2.

To investigate the underlying mechanism of biogenesis of tRFs, which remains largely unknown, the relative abundance of different tRNA-related fragments’ size (15–30 nt) was profiled across tumor types (Figure 2A) and related normal samples (Supplementary Figure S2A). Strikingly, marked size preference was observed in that mature tRNAs were predominantly processed into 18–20, 23 and 30 nt 5′-tRFs and into 16–18, 22 and 24–25 nt 3′-tRFs. On average, these three size ‘peaks’ accounted for 64.6–85.1% (54.1–85.7% in normal specimens) and 87.2–96.8% (90.6–97% in normal specimens) of total abundance among 15 cancer types for 5′-tRFs and 3′-tRFs, respectively. It was also revealed that different tissues and tumor types showed a similar cleavage pattern with three size ‘peaks’ for both 5′-tRFs and 3′-tRFs. A quantitative analysis of the pattern of tRNA cleavage across tRNA genes, tumor types and tissues was carried out using an entropy-based JS cleavage score (26). A higher JS cleavage score generally indicates a more specific cleavage pattern, and conversely, a lower score represents greater similarity. As expected, the JS cleavage score for the majority of tRNA cleavage across cancer types (median of JS cleavage score: 0.22 and 0.19 for 5′-tRFs and 3′-tRFs, respectively) and tissues (median of JS cleavage score: 0.28 and 0.20 for 5′-tRFs and 3′-tRFs, respectively) was found to be relatively low (Figure 2B; Supplementary Figure S2B). This suggests that the cleaved positions of tRNAs are conserved among different tissues and tumor types. Nonetheless, the cleavage pattern among different tRNA types was found to be relatively specific (median of JS cleavage score: 0.86 and 0.71 in tumors for 5′-tRFs and 3′-tRFs, respectively; 0.85 and 0.71 in normal tissues for 5′-tRFs and 3′-tRFs, respectively) (Figure 2B; Supplementary Figure S2B), indicating that the cleaved sites of tRNAs are highly dependent on tRNA families.

To investigate the extent of distinct specific cleavage of different tRNA types, we constructed a landscape showing primary cleaved positions in tRNAs based on relative abundance of each size (Figure 2C and D; Supplementary Figure S2C and D). Overall, it appears that 66.1% and 60.5% of all tRNAs in GtRNAdb can generate 5′-tRFs and 3′-tRFs from their 5′ and 3′ ends, respectively (36). When these tRNAs were ordered using a *k*-means clustering, hotspot sites of cleavage were found in the 5′ or 3′ ends in the majority of these tRNAs (73.7% and 54.7%, respectively). The median JS cleavage score was 0.61 and 0.67 in their 5′ and 3′ ends, respectively, for tumors (Figure 2C and D). For normal tissues, similar cleavage trends were observed (Supplementary Figure S2C and D). Moreover, it was found that these cleavage hotspots differed among tRNA types, whereas they were consistent with the above identified three ‘peaks’ and highly conserved across cancer types and related normal tissues. However, it was also observed that patterns of cleavage of some tRNAs vary in a tumor type-dependent manner. For example, tRNA Asn-GTT-6-1 specifically produced 18 nt fragments from its 5′ ends in head and neck squamous cell carcinoma (HNSC) (Figure 2C). Together, these findings confirm that tRNAs are specifically cleaved and these sites of cleavage are highly dependent on tRNA types.

An independent Db-PCR validation was then performed for two tRNAs in lung cancer tissues. It was found that tRNA^ArgACG-2-1^ produced 5′-tRFs and tRNA^tRX-000020^ produced 3′-tRFs, respectively. As inferred from both TCGA and our own lung cancer dataset, the predominantly cleaved sites of these two tRNAs are 19 nt relative to 5′ end of tRNA^ArgACG-2-1^ and 18 nt relative to 3′ end of tRNA^tRX-000020^, respectively (Figure 2E and F; Supplementary Table S2). The Db-PCR results confirmed our findings that those fragments derived from the predicted major position of cleavage (5′-ArgACG-2-1-L19 and 3′-tRX-000020-L18) are of much higher abundance than fragments from the minor sites of cleavage (Figure 2G and H). To further rule out the potential confounding effect of tRNA modifications on primary cleaved positions in tRNAs, total tumor RNAs were pretreated with an rtStar™ tRF&tiRNA Pretreatment Kit (Arraystar, USA) to demethylate m1A, m1G and m3C. Db-PCR analysis was then performed on these pretreated RNA samples. The distribution of tRF fragments was similar to that observed in untreated samples, confirming cleavage specificity of tRNAs (Figure 2G and H; Supplementary Figure S2E and F). Taken together, our evidence suggests that tRF processing is under tight cellular regulation.

### The dysregulated expression of tRFs in cancer

**Figure 3.**
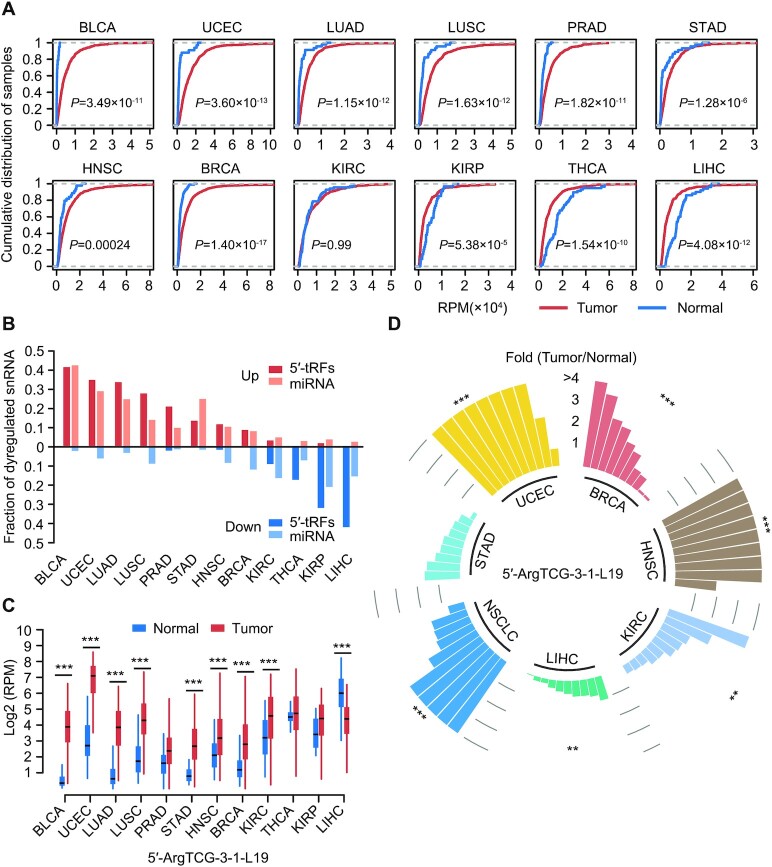
The dysregulated expression of tRFs in cancer. (**A**) Empirical cumulative distribution plot of the overall 5′-tRF expression levels in tumor and normal specimens across 12 cancer types. Tumor samples except kidney renal clear cell carcinoma (KIRC) exhibited a significant shift (most toward to the right side) relative to normal samples. A two-sided Student’s *t*-test was used to calculate *P*-values and assess the significance of the shift of the cumulative distribution curve of the overall 5′-tRF expression levels in tumors relative to normal samples. The overall 5′-tRF expression levels in RPM metric are shown on the *x*-axis. (**B**) The percentage of upregulated and downregulated 5′-tRFs and miRNAs across different cancer types. (**C**) Example of a 5′-tRF, 5′-ArgTCG-3-1-L19, dysregulated in most tumor types. (**D**) Db-PCR analyses of 5′-ArgTCG-3-1-L19 in independent cohorts including breast invasive carcinoma, HNSC, KIRC, LIHC, NSCLC and stomach adenocarcinoma. The bars in panel (D) represent fold changes in tRF expression between tumors and their adjacent normal tissues. Each bar represents a patient. *P < 0.05, **P < 0.01 and ***P < 0.001 based on a two-sided Mann–Whitney test. See also Supplementary Figure S3 and Supplementary Table S3.

We evaluated the total 5′-tRF and 3′-tRF expression across 12 solid tumor types for which sufficient (*n* > 15) corresponding normal samples were available (Figure 3A; Supplementary Figure S3A). Strikingly, it was found that a majority of cancer types showed a significant enrichment of 5′-tRF fragments compared with normal tissues, whereas significant depletion of 5′-tRF fragments was observed in kidney renal papillary cell carcinoma (KIRC), thyroid carcinoma (THCA) and liver hepatocellular carcinoma (LIHC) (Figure 3A). Additionally, individual differentially expressed 5′-tRFs and 3′-tRFs were identified across cancer types (Figure 3B; Supplementary Figure S3B; Supplementary Table S3). The cancer types with overenriched 5′-tRFs fragments had a large fraction of significantly upregulated 5′-tRFs (*P*-value <0.001), while the significantly dysregulated 5′-tRFs of other tumor types were mainly downregulated (*P*-value <0.001). The different patterns of 5′-tRFs’ dysregulation may be due to distinct expression levels of endonucleases responsible for 5′-tRF biogenesis, although this remains unknown. Nevertheless, the findings provide a clue for discovery of underlying molecular determinants.

Dysregulation of miRNAs revealed a similar pattern with our identified 5′-tRFs, suggesting a potential relationship of biogenesis between them (Figure 3B). Further analysis of dysregulated 5′-tRFs among different tumor types revealed that ∼80% and ∼50% of these 5′-tRFs were significantly upregulated and downregulated in at least one cancer type (Supplementary Table S3), respectively. This indicates that there is a common and widespread alteration of 5′-tRFs in cancer. The 5′-ArgTCG-3-1-L19 was significantly upregulated in eight cancer types and significantly downregulated in LIHC [false discovery rate (FDR) <0.01, fold change >2; Figure 3C; Supplementary Table S3). Among all tRFs investigated, expression of 5′-ArgTCG-3-1-L19 was dysregulated in the greatest number of cancer types and thus selected for experimental validation. Db-PCR analysis using multiple independent cohorts further confirmed its existence and common dysregulation across tumor types (Figure 3D; Supplementary Figure S3C). Notably, downregulated expression of 5′-ArgTCG-3-1-L19 was also validated, suggesting the robustness of our analysis and supporting the conclusion that it has specific regulatory roles in LIHC.

### tRF expression data reveal KIRC subtypes and improve outcome prediction

**Figure 4.**
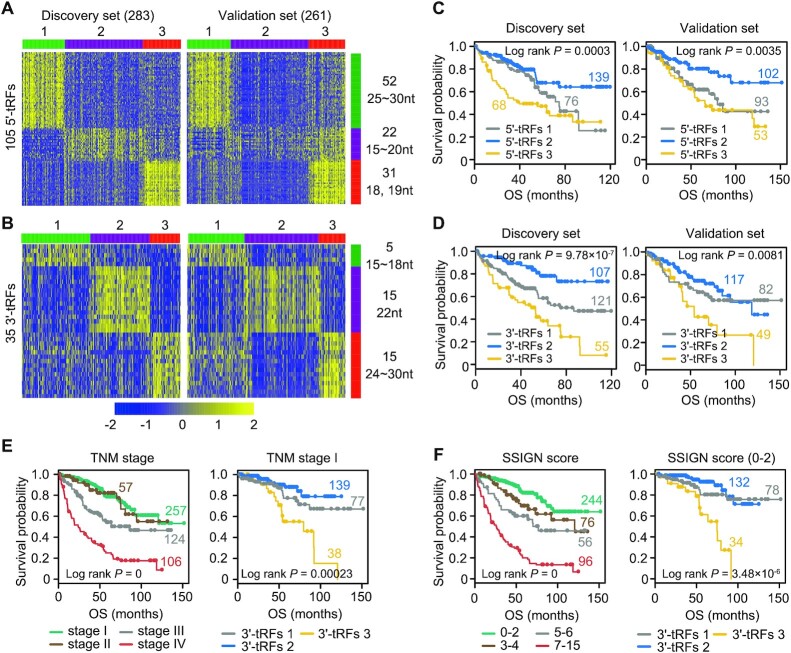
tRF expression data reveal KIRC subtypes and improve outcome prediction. (**A, B**) Tumors were clustered into three subtypes according to tRF expression profiles in a discovery set (left panel). Subtype-specific tRF signatures were extracted and then applied to the validation set, yielding similar three separated groups (right panel). The number and length range of representative tRF features are shown on the right side of the figure. The scale bar indicates tRF expression after standardization. Kaplan–Meier survival analysis showing significant prognostic differences among 5′-tRF (**C**) or 3′-tRF (**D**) expression subtypes in both discovery (left panel) and validation (right panel) sets. *P*-value was calculated by a log-rank test. (**E**) The 3′-tRF expression subtypes could further stratify patients with TNM stage I into groups with distinct prognosis (right panel). Kaplan–Meier plot of the four TNM stages is also shown in the left panel. (**F**) The 3′-tRF expression subtypes could efficiently separate patients with lower Mayo stage, size, grade and necrosis (SSIGN) score into groups with distinct clinical outcome (right panel). Kaplan–Meier plot of the four SSIGN groups is also shown in the left panel. See also Supplementary Figure S4 and Supplementary Table S4.

A total of 544 KIRC samples in the TCGA were sequenced in two different platforms (283 patients using Genome Analyzer IIx and 261 patients using HiSeq 2000), which allowed us to naturally treat them as discovery (*n* = 283) and validation (*n* = 261) sets, respectively. KIRC samples were thus chosen as an example to demonstrate the clinical relevance of tRF expression to patient outcome (see Supplementary Methods). There were no significant differences in clinical characteristics of patients between the discovery and validation sets (Supplementary Table S4A). Analyses of 5′-tRF and 3′-tRF expression profiles in the discovery set both yielded three stable subgroups using a non-negative matrix factorization (NMF) consensus clustering approach (Figure 4A and B). Then, 105 and 35 subtype-specific signatures were identified for all three 5′-tRF- and 3′-tRF-based subtypes, respectively, on the basis of a gene scoring schema. It was thereby found that subtype-specific tRFs for each group were characterized by a given size range. This suggests that the tRFs with different lengths belong to diverse classes of small ncRNAs and may therefore exhibit distinct features of biogenesis and function. The three clusters emerged again when the same ordering of subtype-specific signatures from the discovery set was applied to the validation set (Figure 4A and B). Notably, the percentage of samples in each subtype was similar between the discovery and validation sets. Furthermore, 5′-tRF-based and 3′-tRF-based clusters showed statistically significant differences of survival in both discovery and validation sets (Figure 4C and D). Significant prognostic stratification of subtypes for other cancer types was also observed (Supplementary Figure S4A).

To further evaluate the clinical value of tRF-based molecular subtypes, a multivariate Cox model analysis was performed and adjusted for the American Joint Committee on Cancer (AJCC) TNM stage grouping and the Mayo SSIGN score, respectively. These are commonly used for prognosis of surgically treated KIRC patients. It was found that the risk of patients of 3′-tRF-based subtype 3 was twice that of patients in 3′-tRF-based subtype 2 even after adjusting for the AJCC TNM or the SSIGN score [HR = 2.28 (1.52–3.43) and 2.03 (1.33–3.09), respectively; *P* = 7.23 × 10^−5^ and 0.00099, respectively; Supplementary Table S4B]. Importantly, patients in the AJCC TNM early stage or with a low SSIGN score could be further stratified based on 3′-tRF expression subtypes (Figure 4E and F; Supplementary Figure S4B). These results indicate that 3′-tRF-based subtypes can provide independent prognostic information for KIRC patients. Generally, 5′-tRFs offer less prognostic values for KIRC patients than 3′-tRFs (Supplementary Figure S4C; Supplementary Table S4B).

### Identification of biologically distinct supercluster via cluster analysis of tRF expression subtypes across 15 cancer types

**Figure 5.**
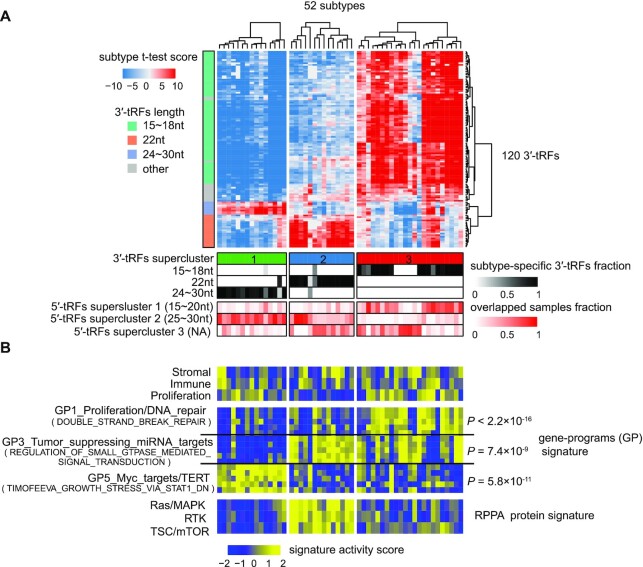
Identification of biologically distinct supercluster via cluster analysis of 3′-tRF expression subtypes across 15 cancer types. (**A**) Unsupervised hierarchical clustering of 52 3′-tRF pan-cancer subtypes identified three superclusters highly correlated with the length of 3′-tRFs. Heatmap represented *t*-test score comparing the 3′-tRF expression level of each 3′-tRF expression subtype with that of other subgroups within the same cancer type. Below the heatmap (top to bottom): group identification numbers of supercluster; the fraction of subtype-specific 3′-tRF signatures belonging to a given group defined by length of 3′-tRFs (see Supplementary Table S5); overlapped fraction between samples of 5′-tRF supercluster and 3′-tRF supercluster. Left side bar shows the length of 3′-tRFs. (**B**) Biologically distinct characterization among three 3′-tRF superclusters. Heatmap reflected *t*-test statistic comparing the single-sample gene set enrichment analysis (ssGSEA) score for gene/protein signatures of each 3′-tRF expression subtype with that of other subgroups within the same cancer type. Below the heatmap (top to bottom): the stroma, immune and proliferation signatures (top panel); 3300 bimodal gene signatures (middle panel), grouped into 22 non-redundant *gene programs* (GPs) symbolizing most cancer hallmarks; RPPA protein signatures (bottom panel). The most correlated GP with each 3′-tRF supercluster was selected according to the enrichment analysis. See also Supplementary Figure S5, Supplementary Table S5 and Supplementary Methods for details on clustering.

To evaluate whether the length of tRFs was also a critical determinant of tRF-based subtypes for other cancer types as found above in KIRC, an NMF clustering analysis was performed using tRF expression profiles and subtype-specific signatures were then determined across 14 other cancer types (see Supplementary Methods). This analysis found that the 3′-tRF expression subtypes for other cancer types were also predominantly determined by the three classes of 3′-tRF size, 15–18 nt (termed 18 nt 3′-tRFs hereafter), precisely 22 nt (termed 22 nt 3′-tRF hereafter) and >24 nt (termed 24nt 3′-tRF hereafter; Supplementary Table S5). To identify common biological processes across 3′-tRF expression subtypes, a pan-cancer analysis was carried out on 52 3′-tRF-based subtypes containing 120 3′-tRFs that existed in at least 13 cancer types. Unsupervised clustering analysis of these 52 tumor subtypes then gave rise to three ‘clusters of clusters’ that we refer to as a supercluster independent of tumor tissue of origin (Figure 5A; Supplementary Table S5). Importantly, the three 3′-tRF-based superclusters, that is 3′-tRF superclusters 1, 2 and 3, including at least 14 types of tumor origins for each supercluster, were characterized by 24, 22 and 18 nt 3′-tRFs, respectively (Figure 5A; Supplementary Table S5). These findings indicate that the size-determined feature of tRF expression subtypes is largely conserved across different cancer types. Moreover, three superclusters were observed in terms of 5′-tRFs. Specifically, 5′-tRF supercluster 1 was mainly driven by 15–20 nt 5′-tRFs (termed 20 nt 5′-tRFs), 5′-tRF supercluster 2 by 5′-tRFs with >25 nt (termed 25 nt 5′-tRFs) and 5′-tRF supercluster 3 has a relatively weak feature of length (Supplementary Figure S5; Supplementary Table S5). These two classes of superclusters were closely correlated with each other in terms of fragment sizes. For example, samples of 3′-tRF supercluster 1 (determined by 24 nt 3′-tRFs) had a large fraction of overlap with that of 5′-tRF supercluster 2 (determined by 25 nt 5′-tRFs) and 3′-tRF supercluster 3 (determined by 18 nt 3′-tRFs) tended to overlap with 5′-tRF supercluster 1 (determined by 20 nt 5′-tRFs) (Figure 5A; Supplementary Figure S5). These results suggest that a similar or the same endonuclease is likely responsible for 24 nt 3′-tRFs and 25 nt 5′-tRFs, while both 18 nt 3′-tRFs and 20 nt 5′-tRFs are likely cleaved by another enzyme or group of similar enzymes.

An ssGSEA was then performed to detect cancer-associated modules of each supercluster characteristic of multiple cross-tissue groups of origin (37). First, 3300 bimodal sets of gene signatures/modules were selected to comprehensively characterize each supercluster (see Supplementary Methods; Supplementary Table S5). These had previously been used to study pan-cancer mRNA expression subtypes (38). These gene signatures were further grouped into 22 non-redundant GPs, which typified a number of cancer hallmarks. Next, an activation score of each signature across samples was calculated by ssGSEA. Finally, gene signature activity for each subtype was measured based on a *t*-test metric that compared the average signature activation score of each subgroup with that of other clusters within the same cancer type. These gene signatures were then sorted in descending order on the basis of 25th quantile of signature activity *t*-test scores across all subtypes within each supercluster. The most highly enriched GP was determined from the top 50 gene signatures. As shown in Figure 5B, 3′-tRF superclusters 1, 2 and 3 were mainly characterized by *GP5_Myc_targets/TERT*, *GP3_Tumor_suppressing_miRNA_targets* and *GP1_Proliferation/DNA_repair*, respectively. Notably, representative signatures of 3′-tRF supercluster 2 were exactly 22 nt, the length of a majority of mature miRNAs. The same or similar characteristics of function and structure between miRNAs and these 3′-tRF signatures suggest that these 22 nt 3′-tRFs may mediate post-transcriptional silencing regulation in multiple cancer types (39).

Pathway scores of each sample were further determined using proteomic data of RPPA (38). The RPPA pathway activity of each subtype was measured based on the approach described earlier. Interestingly, it was shown that Ras/MAPK signaling and the RTK and TSC/mTOR pathway were highly activated in 3′-tRF supercluster 2. In addition, we observed weak correlations between 3'-tRFs and their corresponding precursor tRNAs (*r* = 0.17, *P*-value = 0.03), suggesting that the generation of different tRFs is somehow regulated and therefore a specific feature of different cancer subtypes. Detailed results on the molecular characterization of 5′-tRF superclusters are given in Supplementary Figure S5 and Supplementary Table S5. Overall, the length of tRFs plays a critical role in the formation of tRF expression subtypes and these cross-tumor tRF subtypes are characteristic of common cancer-related processes, providing a basic resource to unify cancer research and therapy.

### Discovery of cancer driver tRFs using an effective approach for accurately exploring cross-cancer and platform trends

**Figure 6.**
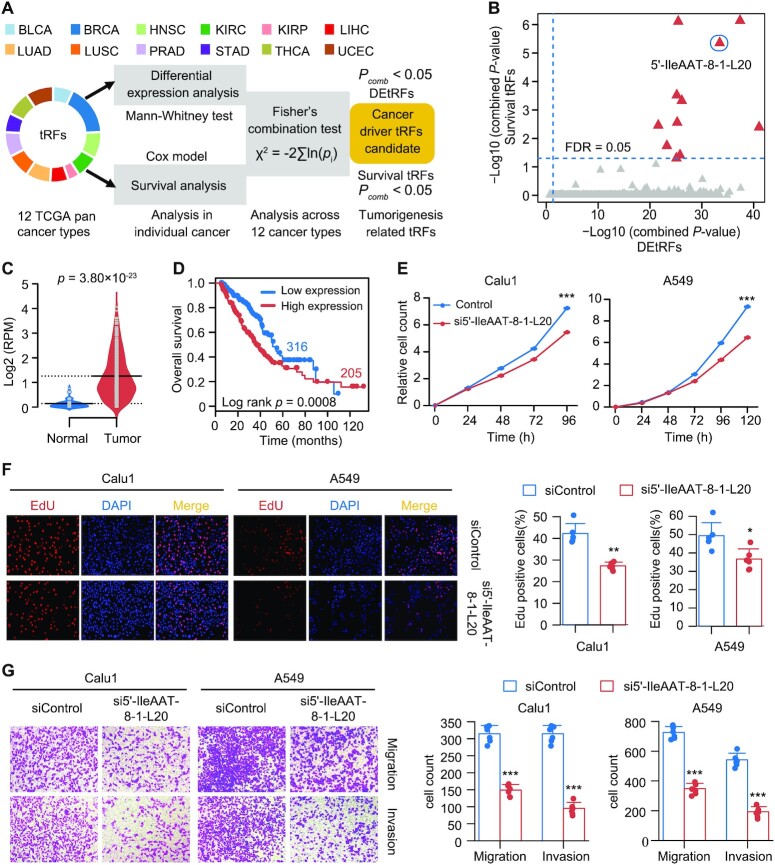
Discovery of cancer driver tRFs using an effective approach for accurately exploring cross-cancer and platform trends. (**A**) Workflow of identifying tRFs that potentially drive the cancer phenotype. DEtRFs, differentially expressed tRFs; survival tRFs, tRFs significantly associated with patient survival; *P*_comb_, integrated *P*-value using Fisher’s approach. (**B**) Scatter plot showing 11 candidate cancer superdriver tRFs (red triangle). (**C**) The expression level of 5′-IleAAT-8-1-L20 in LUAD (*n* = 521) and normal lung tissues (*n* = 46) from TCGA (bars represent median value; *P*-values are from two-sided Mann–Whitney test). (**D**) Kaplan–Meier survival curves of patients grouped by the expression values of 5′-IleAAT-8-1-L20 in the TCGA LUAD cohort. (**E**) Growth curves of Calu1 and A549 cells transfected with 5′-IleAAT-8-1-L20 siRNA or control siRNA. (**F**) Cell proliferation assay using Edu immunofluorescence in 5′-IleAAT-8-1-L20 knockdown cells or control cells. (**G**) Migration and invasion assays following knockdown of 5′-IleAAT-8-1-L20 in Calu1 and A549 cells. The error bars indicate SD of three independent experiments. *P < 0.05, **P < 0.01 and ***P < 0.001 using a two-sided Student’s *t*-test. See also Supplementary Figure S6.

To examine whether these above identified tRFs could drive tumorigenesis, a mining strategy was established that integrated dysregulation of expression and clinical information of tRFs across different tumor types (Figure 6A; see Supplementary Methods). Since tRFs were overexpressed in most cancer types, the aim was to screen candidate tRFs with oncogenic roles. These candidates were expected to be significantly upregulated in tumors and their high expression was significantly associated with worse prognosis. In total, 11 cancer driver tRFs were identified using this screening method (Figure 6B; Supplementary Table S6). These driver tRFs did not show any strong tissue specificity (JS score ranging from 0.15 to 0.22; Supplementary Table S6), while some of them revealed isodecoder specificity (Supplementary Table S6). A 20 nt 5′-tRF derived from tRNA^IleAAT-8-1^ (i.e. ‘5′-IleAAT-8-1-L20’; Figure 1C) was chosen for further validation because of its unique sequence and upregulation associated with poor prognosis in LUAD (Figure 6C and D) and LUSC (Supplementary Figure S6A and B). Its upregulation in lung tumor tissues was validated in an independent non-small lung cancer cohort using Db-PCR (Supplementary Figure S6C). Additionally, the expression of 5′-IleAAT-8-1-L20 is moderate (median log RPM = 1.3; Supplementary Table S3), comparable with several well-known lung cancer-associated miRNAs (40–43) Next, the cancer-related roles of 5′-IleAAT-8-1-L20 were investigated by loss-of-function (siRNA and antisense inhibitor) and gain-of-function assays (oligonucleotide mimic) in two lung cancer cell lines, Calu1 and A549 (see Supplementary Methods; Supplementary Table S7). The expression level of 5′-IleAAT-8-1-L20 was substantially reduced after siRNA was transfected into cells, whereas the expression of its precursor, tRNA^IleAAT-8-1^, was not significantly changed (Supplementary Figure S6D and E). These data show that siRNA specifically targeted 5′-IleAAT-8-1-L20, which was also confirmed by a luciferase reporter assay (Supplementary Figure S6F). As a result, knockdown of 5′-IleAAT-8-1-L20 significantly suppressed both cell growth and clone formation in the two cell lines tested (Figure 6E; Supplementary Figure S6G and H). Inhibition of cell viability was also demonstrated using the Edu immunofluorescence assay in 5′-IleAAT-8-1-L20 knockdown cell lines (Figure 6F). Inhibition of 5′-IleAAT-8-1-L20 was found to significantly reduce cell migration and invasion in both Calu1 and A549 cell lines compared with control cells (Figure 6G). Overexpression of 5′-IleAAT-8-1-L20 also rescued cell phenotypes in these knockdown cells (Supplementary Figure S6I). Finally, these *in vitro* cell-based results were further confirmed by *in vivo* tumorigenicity assays in nude mice (Supplementary Figure S6J–O).

To rule out potential confounding effects of its precursor on our results, another siRNA was designed for targeting the 3′ end of tRNA^IleAAT-8-1^, which is far away from 5′-IleAAT-8-1-L20 and does not affect the expression of 5′-IleAAT-8-1-L20. Of note, downregulation of the expression of tRNA^IleAAT^ that uniquely generated 5′-IleAAT-8-1-L20 had little effect on cell migration and invasion (Supplementary Figure S6P and Q), suggesting that 5′-IleAAT-8-1-L20 functions independently of its precursor. To further rule out potential siRNA-induced off-target effects, cell lines overexpressing 5′-IleAAT-8-1-L20 mimic were also established. Re-expression of 5′-IleAAT-8-1-L20 significantly promoted lung cancer cell growth compared to control cell lines (Supplementary Figure S6R and S). Collectively, these findings suggested that 5′-IleAAT-8-1-L20 plays an oncogenic role in tumor pathogenesis through promoting tumor cell proliferation, migration and invasion.

### 5′-IleAAT-8-1-L20 regulates the cell cycle and does not function as a miRNA

**Figure 7.**
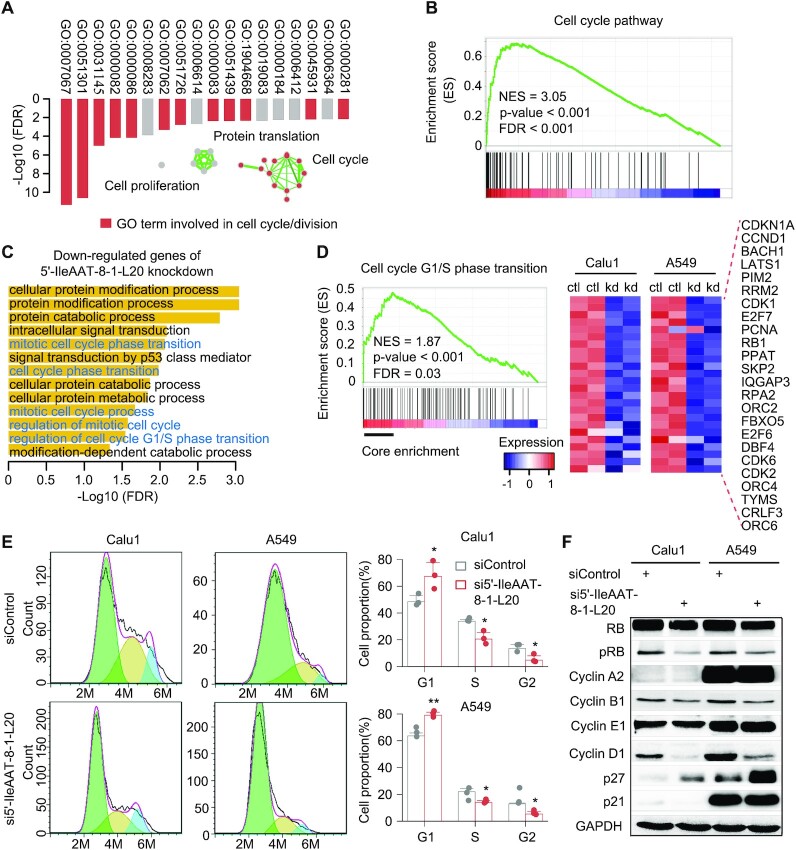
5′-IleAAT-8-1-L20 regulates the cell cycle in a non-miRNA manner. (**A**) Gene ontology of protein-coding genes significantly co-expressed with 5′-IleAAT-8-1-L20 in at least eight cancer types in TCGA. (**B**) Enrichment plot of gene signatures for cell cycle pathway with respect to 5′-IleAAT-8-1-L20 expression level in LUAD. ES, enrichment scores. (**C**) Gene ontology of genes significantly downregulated by knocking down 5′-IleAAT-8-1-L20 in two lung cancer cell lines, Calu1 and A549. Each cell line has two independent replicates. Cell cycle-associated biological processes are shown in blue color. (**D**) Enrichment analysis of gene signatures for cell cycle pathway between control and 5′-IleAAT-8-1-L20 knockdown cell lines: Calu1 and A549. The core enrichment genes within cell cycle-related gene sets are marked by black line (left panel) and their abundance in control and 5′-IleAAT-8-1-L20 knockdown cells is presented using a heatmap plot (right panel). The scale bar represents gene expression after standardization. (**E**) Cell cycle profile of control and 5′-IleAAT-8-1-L20 knockdown cells. *P < 0.05, **P < 0.01 and ***P < 0.001 using a two-sided Student’s *t*-test. (**F**) Western blot analysis of cell cycle-related proteins in control and 5′-IleAAT-8-1-L20 knockdown cells. GAPDH protein was used as a control. Light green, light gold and light blue represent G1, S and G2 + M, respectively. See also Supplementary Figure S7.

Studies of the function of tRFs have been problematic due to a lack of *a priori* knowledge. To determine the functional roles of 5′-IleAAT-8-1-L20, a guilt-by-association analysis based on co-expression patterns was performed, an analysis that has been widely used for studying long ncRNAs (44). This analysis yielded a total of 491 protein-coding genes that were significantly co-expressed with 5′-IleAAT-8-1-L20 in at least eight cancer types. These genes were mainly enriched in cell cycle/division-related biological processes (Figure 7A). Additionally, GSEA also found a significant enrichment of gene signatures in the cell cycle pathway with regard to the expression levels of 5′-IleAAT-8-1-L20 in the TCGA LUAD cohort (Figure 7B) (45). Together, these results suggest a potential role of 5′-IleAAT-8-1-L20 in cell cycle regulation.

To assess this hypothesis, transcriptomic sequencing data from 5′-IleAAT-8-1-L20 knockdown Calu1 and A549 cells or controls were analyzed. Gene Ontology (GO) analysis revealed that these downregulated genes (FDR < 0.05) were significantly correlated with many cell cycle-associated GO terms, such as mitotic cell cycle phase transition and cell cycle phase transition (Figure 7C). GSEA further showed that gene signatures of G1/S phase transition were significantly and negatively enriched in 5′-IleAAT-8-1-L20 knockdown cells (Figure 7D) and the most downregulated genes related with cell cycle regulation are also shown in Figure 7D. The results of sequencing analysis in 5′-IleAAT-8-1-L20 knockdown cells validated our hypothesis. Next, the regulatory consequence of 5′-IleAAT-8-1-L20 on the cell cycle was experimentally explored. Consistent with our earlier hypothesis, knockdown of 5′-IleAAT-8-1-L20 dramatically suppressed the transition from the G1 phase to the S and G2 phases of cell cycle (Figure 7E). Additionally, knockdown of 5′-IleAAT-8-1-L20 significantly affected the expression levels of cell cycle-related proteins, such as cyclin D1, p27 and phosphorylated RB (Figure 7F), further supporting our hypothesis. Overall, these results indicate that 5′-IleAAT-8-1-L20 plays an important role in cell cycle progression and that this hypothesis-driven method can be effectively applied for functional investigation of tRFs.

Depletion of 5′-IleAAT-8-1-L20 induced altered gene expression along with downregulation of cell cycle-related genes as described earlier (Figure 7C–F; Supplementary Figure S7A). Next, we sought to determine whether 5′-IleAAT-8-1-L20 functions similar to miRNAs in regulating gene expression. First, the sequence of 5′-IleAAT-8-1-L20 was systematically blasted in the miRBase (v21). Even allowing more than one mismatch, no similar sequences of miRNA with 5′-IleAAT-8-1-L20 were found. Second, knockdown of 5′-IleAAT-8-1-L20 did not induce significant global upregulation of 5′-IleAAT-8-1-L20 targets, which were predicted in the same way as miRNAs (Supplementary Figure S7B). Third, either overexpression of tRNA^IleAAT-8-1^ or 5′-IleAAT-8-1-L20 mimic did not inhibit the expression of antisense reporter (Supplementary Figure S7C). Finally, analysis of immunoprecipitation experiments (RIP) showed significant enrichment of has-miR-21 in Argonaute protein complexes (33-fold versus control), while no significant enrichment was observed for 5′-IleAAT-8-1-L20 (1.1-fold versus control) (Supplementary Figure S7D and E). We thus concluded that 5′-IleAAT-8-1-L20 likely regulates gene expression in a non-miRNA manner.

## DISCUSSION

Recent discovery of the functional role of several types of tRFs (e.g. tRNA halves, 3′-tRFs and 3′U-tRFs) in tumor pathogenesis prompted us to explore whether other types (e.g. 5′-tRFs) are also implicated in tumorigenesis (18–21). Although those classes of tRFs have been reported in many organisms, any cancer-associated function has remained largely uncharacterized so far. Here, a bioinformatic ‘big data’ approach was taken to functionally and clinically characterize 5′-tRF and 3′-tRF expression profiles in >8000 specimens across 15 tumor types. As far as we know, this represents one of the largest analyses of 5′-tRFs and 3′-tRFs across human cancers. In comparison to previous studies (46–48), our analysis was more robust due to the filtering of samples/cohorts and narrower selection of tRFs (25,49). These findings increase our understanding of the biogenesis, characteristics and functional roles of 5′-tRFs and 3′-tRFs in cancers.

The cross-tumor analysis depicted a genomic landscape of endogenous 5′-tRFs and 3′-tRFs. Through this landscape, we systematically determined that these tRFs are indeed biologically and functionally relevant and not random degraded fragments of tRNAs. This analysis provided the strong evidence that cleavage of tRNA is under tight cellular regulation and the cleaved position is highly conserved among different tissues and tumor types. For example, mature tRNAs were predominantly processed into 16–18, 22 and 24–25 nt 3′-tRFs, accounting for the large majority of 3′-tRFs. These 3′-tRFs are characterized by size-dependent features and functionality. Notably, frequent and widespread dysregulation of the expression of 5′-tRFs and 3′-tRFs was observed at the global and individual levels among most cancer types. Abundance of 5′-tRFs and 3′-tRFs was generally of the same order of magnitude as miRNAs.

Our results revealed significant commonality among tRF expression subtypes from distinct tissues of origins. Three biologically distinct superclusters independent of tumor tissues of origin were identified that were characterized by commonly activated cancer pathways. For example, a group of 3′-tRFs with precise 22 nt was found coincidentally upregulated across a majority of (13/15) cancer types, all of which share the activation of Ras/MAPK, RTK and TSC/mTOR signaling. These findings provide us with the possibility that tRF-targeted therapy developed in one cancer may be extrapolated to other cancer types, which may benefit from their similar molecular patterns.

In addition to these common molecular characteristics across cancers, heterogeneous tRF expression signatures were observed within the same cancer type. For instance, signatures of 18 and 22 nt 3′-tRFs defined distinct tumor subtypes and this pattern was found to be extremely conserved among different cancer types. Of note, the 18 and 22 nt 3′-tRFs have been previously termed as tRF-3a and tRF-3b in many organisms, respectively (32). The findings of this study further suggest that there are distinguishable features of biogenesis and functional performance between the two types of 3′-tRFs, consistent with more recent discoveries that 18 and 22 nt 3′-tRFs suppressed mobility of endogenous retroviruses through distinct regulatory mechanisms in mouse cells (14). The underlying reasons for their differences may be two extra chemical modifications in 22 nt 3′-tRFs. Additionally, 22 nt 3′-tRFs were associated with miRNA-targeted processes, implying that they may be involved in post-transcriptional silencing regulation, as shown in previous studies (7,14,39,50,51).

This study also provided the first dry and wet evidence that 5′-tRFs may play an important role in tumor oncogenesis and progression. 5′-IleAAT-8-1-L20 was found to be a cancer driver tRF through integrative bioinformatic analyses, which was experimentally confirmed showing that inhibition of 5′-IleAAT-8-1-L20 suppresses cell proliferation, migration and invasion in lung cancer cell lines and animal models. Additionally, 5′-IleAAT-8-1-L20 knockdown inhibited cell cycle progression, as predicted by our gene co-expression analyses. The results collectively revealed the oncogenic role of 5′-IleAAT-8-1-L20 in cancer. However, tRNA fragments derived from the other 13 IleAAT isotypes showed no significant differences in expression between tumor and normal tissue and/or no significant relationship with patient survival. It was therefore one specific isodecoder of an IleAAT isoacceptor, not the group of isodecoders, that was associated with lung cancer, which was consistent with previous findings for neuronal homeostasis (52). We detected the protein expression of IIeRS (cytoplasmic) and IIeRS2 (mitochondrial) after knockdown IIeAAT-8-1, and observed that IIeAAT-8-1 silencing has limited effects on IIeRS and IIeRS2 protein expression (Supplementary Figure S8). Further investigations are required to clarify its mechanism of regulating downstream genes.

Several caveats for our study should be acknowledged. First, tRNA halves (mainly in 32–34 nt) (21,22) and ‘internal’ fragments (i.e. i-tRFs) (53) cannot be effectively detected in our analyses since most of the smRNA-seq data were generated by single-end sequencing for 30 cycles in TCGA datasets. Compared to tRNA halves, the biological and clinical importance of 5′- and 3′-tRFs (tRNA fragments <30 nt) in solid tumors remains largely unknown. Thus, we exclusively focused on 5′- and 3′-tRFs (tRNA fragments <30 nt) in our analyses. To avoid overlooking potentially important findings, we included tRFs of 30 nt in our analysis, although these tRFs may be truncated products of longer tRNA halves. To evaluate the potential influence of these ‘30-proxy’ tRFs, we performed additional cluster analysis using tRFs whose lengths were <27 nt (Supplementary Figures S9 and S10). These three superclusters remained unchanged, suggesting that the inclusion of ‘30-proxy’ tRFs in the analysis did not influence our conclusions. Second, the dysregulation of tRNA expression may lead to differences in tRF abundance (54). Thus, it will be necessary to further validate whether the differential expression of tRF candidates depends on the tRNA gene of its origin. Third, our comparison between smRNA-seq and ARM-seq data from human B lymphocyte-derived cell lines showed that there was a strong correlation between the expression levels of tRFs quantified by the two methods. However, we could not exclude the possibility that the detection and quantification of a few tRFs may be affected by heavy tRNA modifications, which can lead to incomplete reverse transcription. Moreover, tRNA modifications are likely to be involved in the formation of tRFs. As our observation indicated (Supplementary Figure S11), there is a significant correlation between tRNA modifiers and tRF expression in a cancer type-specific manner.

In summary, our work reported a functional genomic landscape of tRFs in human cancers and indicates that tRFs play a critical role in tumor pathogenesis. This work increases our understanding of biogenesis, characteristics and function of this class of novel small non-coding tRFs. It also provides a valuable resource, including the detected tRFs, cancer-associated tRFs and tRF tumor subtypes, for future efforts to identify diagnostic and prognostic biomarkers, develop cancer therapy and study cancer pathogenesis. To make our findings available to the research community, we have recently deposited our results into a public database OncotRF (http://bioinformatics.zju.edu.cn/OncotRF/) (23).

## DATA AVAILABILITY

Raw sequencing data generated in this study have been submitted to NCBI Sequence Read Achieve (https://www.ncbi.nlm.nih.gov/sra/) under accession numbers SRP227912 and SRP227900.

## Supplementary Material

zcaa031_Supplemental_Files

## References

[1] Kirchner S., Ignatova Z. Emerging roles of tRNA in adaptive translation, signalling dynamics and disease. Nat. Rev. Genet. 2015; 16:98–112. 25534324 10.1038/nrg3861

[2] Schimmel P. The emerging complexity of the tRNA world: mammalian tRNAs beyond protein synthesis. Nat. Rev. Mol. Cell Biol. 2018; 19:45–58. 28875994 10.1038/nrm.2017.77

[3] Parisien M., Wang X., Pan T. Diversity of human tRNA genes from the 1000-genomes project. RNA Biol. 2013; 10:1853–1867. 24448271 10.4161/rna.27361 PMC3917988

[4] Saikia M., Krokowski D., Guan B.-J., Ivanov P., Parisien M., Hu G.-F., Anderson P., Pan T., Hatzoglou M. Genome-wide identification and quantitative analysis of cleaved tRNA fragments induced by cellular stress. J. Biol. Chem. 2012; 287:42708–42725. 23086926 10.1074/jbc.M112.371799 PMC3522271

[5] Cole C., Sobala A., Lu C., Thatcher S.R., Bowman A., Brown J.W., Green P.J., Barton G.J., Hutvagner G. Filtering of deep sequencing data reveals the existence of abundant Dicer-dependent small RNAs derived from tRNAs. RNA. 2009; 15:2147–2160. 19850906 10.1261/rna.1738409 PMC2779667

[6] Li Z., Ender C., Meister G., Moore P.S., Chang Y., John B. Extensive terminal and asymmetric processing of small RNAs from rRNAs, snoRNAs, snRNAs, and tRNAs. Nucleic Acids Res. 2012; 40:6787–6799. 22492706 10.1093/nar/gks307 PMC3413118

[7] Sobala A., Hutvagner G. Small RNAs derived from the 5′ end of tRNA can inhibit protein translation in human cells. RNA Biol. 2013; 10:553–563. 23563448 10.4161/rna.24285 PMC3710361

[8] Haussecker D., Huang Y., Lau A., Parameswaran P., Fire A.Z., Kay M.A. Human tRNA-derived small RNAs in the global regulation of RNA silencing. RNA. 2010; 16:673–695. 20181738 10.1261/rna.2000810 PMC2844617

[9] Fu H., Feng J., Liu Q., Sun F., Tie Y., Zhu J., Xing R., Sun Z., Zheng X. Stress induces tRNA cleavage by angiogenin in mammalian cells. FEBS Lett. 2009; 583:437–442. 19114040 10.1016/j.febslet.2008.12.043

[10] Lee Y.S., Shibata Y., Malhotra A., Dutta A. A novel class of small RNAs: tRNA-derived RNA fragments (tRFs). Genes Dev. 2009; 23:2639–2649. 19933153 10.1101/gad.1837609 PMC2779758

[11] Chen Q., Yan M., Cao Z., Li X., Zhang Y., Shi J., Feng G.H., Peng H., Zhang X., Zhang Y. et al. Sperm tsRNAs contribute to intergenerational inheritance of an acquired metabolic disorder. Science. 2016; 351:397–400. 26721680 10.1126/science.aad7977

[12] Sharma U., Conine C.C., Shea J.M., Boskovic A., Derr A.G., Bing X.Y., Belleannee C., Kucukural A., Serra R.W., Sun F. et al. Biogenesis and function of tRNA fragments during sperm maturation and fertilization in mammals. Science. 2016; 351:391–396. 26721685 10.1126/science.aad6780 PMC4888079

[13] Zhang Y., Zhang X., Shi J., Tuorto F., Li X., Liu Y., Liebers R., Zhang L., Qu Y., Qian J. Dnmt2 mediates intergenerational transmission of paternally acquired metabolic disorders through sperm small non-coding RNAs. Nat. Cell Biol. 2018; 20:535–540. 29695786 10.1038/s41556-018-0087-2 PMC5926820

[14] Schorn A.J., Gutbrod M.J., LeBlanc C., Martienssen R. LTR-retrotransposon control by tRNA-derived small RNAs. Cell. 2017; 170:61–71. 28666125 10.1016/j.cell.2017.06.013 PMC5551035

[15] Anderson P., Ivanov P.J.F.l tRNA fragments in human health and disease. FEBS Lett. 2014; 588:4297–4304. 25220675 10.1016/j.febslet.2014.09.001 PMC4339185

[16] Zhu L., Ge J., Li T., Shen Y., Guo J.J.C.L. tRNA-derived fragments and tRNA halves: the new players in cancers. Cancer Lett. 2019; 452:31–37. 30905816 10.1016/j.canlet.2019.03.012

[17] Polacek N., Ivanov P. The regulatory world of tRNA fragments beyond canonical tRNA biology. RNA Biol. 2020; 17:1057–1059. 32715957 10.1080/15476286.2020.1785196 PMC7549696

[18] Goodarzi H., Liu X., Nguyen H.C., Zhang S., Fish L., Tavazoie S.F. Endogenous tRNA-derived fragments suppress breast cancer progression via YBX1 displacement. Cell. 2015; 161:790–802. 25957686 10.1016/j.cell.2015.02.053 PMC4457382

[19] Honda S., Loher P., Shigematsu M., Palazzo J.P., Suzuki R., Imoto I., Rigoutsos I., Kirino Y. Sex hormone-dependent tRNA halves enhance cell proliferation in breast and prostate cancers. Proc. Natl Acad. Sci. U.S.A. 2015; 112:E3816–E3825. 26124144 10.1073/pnas.1510077112 PMC4517238

[20] Balatti V., Nigita G., Veneziano D., Drusco A., Stein G.S., Messier T.L., Farina N.H., Lian J.B., Tomasello L., Liu C.G. et al. tsRNA signatures in cancer. Proc. Natl Acad. Sci. U.S.A. 2017; 114:8071–8076. 28696308 10.1073/pnas.1706908114 PMC5544330

[21] Pekarsky Y., Balatti V., Palamarchuk A., Rizzotto L., Veneziano D., Nigita G., Rassenti L.Z., Pass H.I., Kipps T.J., Liu C.G. et al. Dysregulation of a family of short noncoding RNAs, tsRNAs, in human cancer. Proc. Natl Acad. Sci. U.S.A. 2016; 113:5071–5076. 27071132 10.1073/pnas.1604266113 PMC4983805

[22] Kim H.K., Fuchs G., Wang S., Wei W., Zhang Y., Park H., Roy-Chaudhuri B., Li P., Xu J., Chu K. et al. A transfer-RNA-derived small RNA regulates ribosome biogenesis. Nature. 2017; 552:57–62. 29186115 10.1038/nature25005 PMC6066594

[23] Yao D., Sun X., Zhou L., Amanullah M., Pan X., Liu Y., Liang M., Liu P., Lu Y. OncotRF: an online resource for exploration of tRNA-derived fragments in human cancers. RNA Biol. 2020; 17:1081–1091. 32597311 10.1080/15476286.2020.1776506 PMC7577240

[24] Li H., Durbin R. Fast and accurate short read alignment with Burrows–Wheeler transform. Bioinformatics. 2009; 25:1754–1760. 19451168 10.1093/bioinformatics/btp324 PMC2705234

[25] De Rie D., Abugessaisa I., Alam T., Arner E., Arner P., Ashoor H., Åström G., Babina M., Bertin N., Burroughs A.M. An integrated expression atlas of miRNAs and their promoters in human and mouse. Nat. Biotechnol. 2017; 35:872–878. 28829439 10.1038/nbt.3947 PMC5767576

[26] Cabili M.N., Trapnell C., Goff L., Koziol M., Tazon-Vega B., Regev A., Rinn J.L. Integrative annotation of human large intergenic noncoding RNAs reveals global properties and specific subclasses. Genes Dev. 2011; 25:1915–1927. 21890647 10.1101/gad.17446611 PMC3185964

[27] Kawaji H., Nakamura M., Takahashi Y., Sandelin A., Katayama S., Fukuda S., Daub C.O., Kai C., Kawai J., Yasuda J. et al. Hidden layers of human small RNAs. BMC Genomics. 2008; 9:157. 18402656 10.1186/1471-2164-9-157 PMC2359750

[28] Cozen A.E., Quartley E., Holmes A.D., Hrabeta-Robinson E., Phizicky E.M., Lowe T.M. ARM-seq: AlkB-facilitated RNA methylation sequencing reveals a complex landscape of modified tRNA fragments. Nat. Methods. 2015; 12:879–884. 26237225 10.1038/nmeth.3508 PMC4553111

[29] Telonis A.G., Loher P., Magee R., Pliatsika V., Londin E., Kirino Y., Rigoutsos I. tRNA fragments show intertwining with mRNAs of specific repeat content and have links to disparities. Cancer Res. 2019; 79:3034–3049. 30996049 10.1158/0008-5472.CAN-19-0789 PMC6571059

[30] Honda S., Kirino Y. Dumbbell-PCR: a method to quantify specific small RNA variants with a single nucleotide resolution at terminal sequences. Nucleic Acids Res. 2015; 43:e77. 25779041 10.1093/nar/gkv218 PMC4499115

[31] Gebetsberger J., Polacek N. Slicing tRNAs to boost functional ncRNA diversity. RNA Biol. 2013; 10:1798–1806. 24351723 10.4161/rna.27177 PMC3917982

[32] Kumar P., Anaya J., Mudunuri S.B., Dutta A. Meta-analysis of tRNA derived RNA fragments reveals that they are evolutionarily conserved and associate with AGO proteins to recognize specific RNA targets. BMC Biol. 2014; 12:78. 25270025 10.1186/s12915-014-0078-0 PMC4203973

[33] Martinez G., Choudury S.G., Slotkin R.K. tRNA-derived small RNAs target transposable element transcripts. Nucleic Acids Res. 2017; 45:5142–5152. 28335016 10.1093/nar/gkx103 PMC5605234

[34] Selitsky S.R., Baran-Gale J., Honda M., Yamane D., Masaki T., Fannin E.E., Guerra B., Shirasaki T., Shimakami T., Kaneko S. et al. Small tRNA-derived RNAs are increased and more abundant than microRNAs in chronic hepatitis B and C. Sci. Rep. 2015; 5:7675. 25567797 10.1038/srep07675 PMC4286764

[35] Djebali S., Davis C.A., Merkel A., Dobin A., Lassmann T., Mortazavi A., Tanzer A., Lagarde J., Lin W., Schlesinger F. et al. Landscape of transcription in human cells. Nature. 2012; 489:101–108. 22955620 10.1038/nature11233 PMC3684276

[36] Chan P.P., Lowe T.M. GtRNAdb 2.0: an expanded database of transfer RNA genes identified in complete and draft genomes. Nucleic Acids Res. 2016; 44:D184–D189. 26673694 10.1093/nar/gkv1309 PMC4702915

[37] Martinez E., Yoshihara K., Kim H., Mills G.M., Trevino V., Verhaak R.G. Comparison of gene expression patterns across 12 tumor types identifies a cancer supercluster characterized by TP53 mutations and cell cycle defects. Oncogene. 2015; 34:2732–2740. 25088195 10.1038/onc.2014.216 PMC4317393

[38] Hoadley K.A., Yau C., Wolf D.M., Cherniack A.D., Tamborero D., Ng S., Leiserson M.D.M., Niu B., McLellan M.D., Uzunangelov V. et al. Multiplatform analysis of 12 cancer types reveals molecular classification within and across tissues of origin. Cell. 2014; 158:929–944. 25109877 10.1016/j.cell.2014.06.049 PMC4152462

[39] Maute R.L., Schneider C., Sumazin P., Holmes A., Califano A., Basso K., Dalla-Favera R. tRNA-derived microRNA modulates proliferation and the DNA damage response and is down-regulated in B cell lymphoma. Proc. Natl Acad. Sci. U.S.A. 2013; 110:1404–1409. 23297232 10.1073/pnas.1206761110 PMC3557069

[40] Zhang W.C., Chin T.M., Yang H., Nga M.E., Lunny D.P., Lim E.K.H., Sun L.L., Pang Y.H., Leow Y.N., Malusay S.R.Y. Tumour-initiating cell-specific miR-1246 and miR-1290 expression converge to promote non-small cell lung cancer progression. Nat. Commun. 2016; 7:11702. 27325363 10.1038/ncomms11702 PMC4919505

[41] Wang Y., Xu L., Jiang L. miR-1271 promotes non-small-cell lung cancer cell proliferation and invasion via targeting HOXA5. Biochem. Biophys. Res. Commun. 2015; 458:714–719. 25686496 10.1016/j.bbrc.2015.02.033

[42] Zhou Z., Niu X., Li C., Sheng S., Lu S. Inhibition of the growth of non-small cell lung cancer by miRNA-1271. Am. J. Transl. Res. 2015; 7:1917. 26692935 PMC4656768

[43] Gao Y., Xue Q., Wang D., Du M., Zhang Y., Gao S. miR-873 induces lung adenocarcinoma cell proliferation and migration by targeting SRCIN1. Am. J. Transl. Res. 2015; 7:2519–2526. 26807196 PMC4697728

[44] Huarte M., Guttman M., Feldser D., Garber M., Koziol M.J., Kenzelmann-Broz D., Khalil A.M., Zuk O., Amit I., Rabani M. A large intergenic noncoding RNA induced by p53 mediates global gene repression in the p53 response. Cell. 2010; 142:409–419. 20673990 10.1016/j.cell.2010.06.040 PMC2956184

[45] 3Subramanian A., Tamayo P., Mootha V.K., Mukherjee S., Ebert B.L., Gillette M.A., Paulovich A., Pomeroy S.L., Golub T.R., Lander E.S. et al. Gene set enrichment analysis: a knowledge-based approach for interpreting genome-wide expression profiles. Proc. Natl Acad. Sci. U.S.A. 2005; 102:15545–15550. 16199517 10.1073/pnas.0506580102 PMC1239896

[46] Pliatsika V., Loher P., Magee R., Telonis A.G., Londin E., Shigematsu M., Kirino Y., Rigoutsos I. MINTbase v2.0: a comprehensive database for tRNA-derived fragments that includes nuclear and mitochondrial fragments from all The Cancer Genome Atlas projects. Nucleic Acids Res. 2018; 46:D152–D159. 29186503 10.1093/nar/gkx1075 PMC5753276

[47] Pliatsika V., Loher P., Telonis A.G., Rigoutsos I. MINTbase: a framework for the interactive exploration of mitochondrial and nuclear tRNA fragments. Bioinformatics. 2016; 32:2481–2489. 27153631 10.1093/bioinformatics/btw194 PMC4978933

[48] Zheng L.L., Xu W.L., Liu S., Sun W.J., Li J.H., Wu J., Yang J.H., Qu L.H. tRF2Cancer: a web server to detect tRNA-derived small RNA fragments (tRFs) and their expression in multiple cancers. Nucleic Acids Res. 2016; 44:W185–W193. 27179031 10.1093/nar/gkw414 PMC4987945

[49] Yan X., Hu Z., Feng Y., Hu X., Yuan J., Zhao Sihai D., Zhang Y., Yang L., Shan W., He Q. et al. Comprehensive genomic characterization of long non-coding RNAs across human cancers. Cancer Cell. 2015; 28:529–540. 26461095 10.1016/j.ccell.2015.09.006 PMC4777353

[50] Keam S.P., Sobala A., Ten Have S., Hutvagner G. tRNA-derived RNA fragments associate with human multisynthetase complex (MSC) and modulate ribosomal protein translation. J. Proteome Res. 2017; 16:413–420. 27936807 10.1021/acs.jproteome.6b00267

[51] Kuscu C., Kumar P., Kiran M., Su Z., Malik A., Dutta A. tRNA fragments (tRFs) guide Ago to regulate gene expression post-transcriptionally in a Dicer-independent manner. RNA. 2018; 24:1093–1105. 29844106 10.1261/rna.066126.118 PMC6049499

[52] Ishimura R., Nagy G., Dotu I., Zhou H., Yang X.-L., Schimmel P., Senju S., Nishimura Y., Chuang J.H., Ackerman S.L. Ribosome stalling induced by mutation of a CNS-specific tRNA causes neurodegeneration. Science. 2014; 345:455–459. 25061210 10.1126/science.1249749 PMC4281038

[53] Telonis A.G., Loher P., Honda S., Jing Y., Palazzo J., Kirino Y., Rigoutsos I. Dissecting tRNA-derived fragment complexities using personalized transcriptomes reveals novel fragment classes and unexpected dependencies. Oncotarget. 2015; 6:24797–24822. 26325506 10.18632/oncotarget.4695 PMC4694795

[54] Torres A.G., Reina O., Attolini C.S.-O., de Pouplana L.R. Differential expression of human tRNA genes drives the abundance of tRNA-derived fragments. Proc. Natl Acad. Sci. U.S.A. 2019; 116:8451–8456. 30962382 10.1073/pnas.1821120116 PMC6486751

