# Supplementary material for: Global identification and characterization of tRNA-derived RNA fragment landscapes across human cancers
Source: NAR Cancer. 2020 Oct 19;2(4):zcaa031. doi: 10.1093/narcan/zcaa031 (PMC8210304; doi:10.1093/narcan/zcaa031)
Supplement: zcaa031_Supplemental_Files [file zcaa031_supplemental_files.zip › Supplemental-Materials-R1.pdf]

Global Identification and Characterization of tRNA-Derived RNA Fragment Landscapes across Human Cancers

Xiwei Sun, Juze Yang, Mengqian Yu, Dongxia Yao, Liyuan Zhou, Xufan Li, Qiongzi Qiu, Weiqiang Lin, Bingjian Lu, Enguo Chen, Ping Wang, Wantao Chen, Sifeng Tao, Haiming Xu, Anna Williams, Yong Liu, Xiaoqing Pan, Allen W. Cowley, Jr. , Weiguo Lu, Mingyu Liang, Pengyuan Liu, Yan Lu

Supplementary Data

Supplementary Methods ……………………………………………………….......4

Subcellular localization of tRFs……………………………………………………………………….4

Tissue specimens…………………………………………………...…………………………………4

Jensen Shannon divergence (JS) cleavage score……………………………………………………4

Tissue specificity (TS) score……………………………………………………………………….....5

Differential expression analysis………………………………………………………………………6

Survival analysis in kidney cancer……………………………………………………………………6

Identification of tumor subtypes based on tRF expression…………………………………………6

Identification of supercluster……………………………………..…………………………………..6

Single sample gene set enrichment analysis (ssGSEA)……………………………………………7

Pathway score using RPPA……………………………..……………………………………………7

Identification of cancer driver tRFs…………………………… ……………………………….7

GO analysis………………………….……………………………………………………………….. 7

GSEA analysis…………………………………………………………………………………..….8

Dumbbell-PCR for tRFs and four-leaf clover qRT-PCR for tRNAs………………………………8

Northern Blot……………………………………………………………………………………………8

RPPA protein data…………………………………………………………………………….………8

miRNA-seq data……...………………………………………………………………….………9

mRNA-seq data………………………………………………………………………………….………9

Clinical data of TCGA patients……………………………………………………………….………9

Cell culture……………………………………………………... ……………………….………9

Exogenous tRF and siRNA transfection……………………………………………………………9

Cell proliferation assay…………………………………………………….…………………….………9

Clone formation assay……………………………………………..……………….…..…10

Edu incorporation assay………………………………………………………………...……10

Migration and invasion assay…………………………………………………………...……10

Cell Cycle assay……………………… ………….……………………………….………10

Western blotting……………………… ……….…………………………………….………10

Immunoprecipitation experiment (RIP) assay… ……………………………………….………10

Luciferase reporter assay………………………………………………………………….………11

Animal experiments………………………………………………………………………………11

Supplementary Figures ………….. ………………………………………………….11

Supplementary Fig. S1, related to Fig. 1. Identification and quantification of endogenous tRFs across 15 cancer types. ………………………………………………………….……………..12

Supplementary Fig. S2, related to Fig. 2. tRFs resulted from the specific cleavage of tRNAs in cancer. ………………………………………………………………………………….………14

Supplementary Fig. S3, related to Fig. 3. The dysregulated expression of tRFs in Cancer……..15

Supplementary Fig. S4, related to Fig. 4. tRF expression data reveal KIRC subtypes and improve outcome prediction…………………………………………………………………………..16

Supplementary Fig. S5, related to Fig. 5. Identification of biologically distinct supercluster via cluster analysis of 5′-tRF expression subtypes across 15 cancer types……………………………18

Supplementary Fig. S6, related to Fig. 6. Discovery of cancer driver tRFs using an effective approach for accurately exploring cross-cancer and platform trends……………………………..19

Supplementary Fig. S7, related to Fig. 7. 5′-IleAAT-8-1-L20 regulates the cell cycle in a non-miRNA manner………………………………….. ……………………………………………22

Supplementary Fig. S8, Effect of knockdown of IIeAAT-8-1 on IIeRS and IIeRS2 protein expression ……………………………………….. ……………………………………………23

Supplementary Fig. S9. Identification of biologically distinct supercluster via cluster analysis of 3′-tRF expression subtypes (after removing ambiguous tRFs and tRFs with >27nt) across 15 cancer types………………………………………………………………………………………24

Supplementary Fig. S10. Identification of biologically distinct supercluster via cluster analysis of 5′-tRF expression subtypes (after removing ambiguous tRFs and tRFs with >27nt) across 15 cancer types……...……………………………………………………………………………25

Supplementary Fig. S11. Association of tRNA modification enzymes with 5′-tRF from nucleus, mitochondria, 3′-tRF from nucleus and mitochondria across cancer types ……………………..26

Supplementary Tables………… ……………………………………………………..27

Supplementary Table S1, related to Fig. 1. Summary of TCGA samples used in this study and summary of 5′-tRFs, 3′-tRFs and 3′U-tRFs identified in this study……………………………..27

Supplementary Table S2, related to Fig. 2. Summary of total reads mapped to 5′-tRFs, 3′-tRFs and 3′U-tRFs in our miRNA-seq data set of 18 lung tumors and matched normal tissues from non-small cell lung cancer patients…………………………………………….……………………27

Supplementary Table S3, related to Fig. 3. Summary of dysregulated 5′-tRFs and 3′-tRFs across different cancer types……………………………………………………………………………..27

Supplementary Table S4, related to Fig. 4. (A) Clinical characteristic analyses of KIRC patients in TCGA. (B) Cox analyses of tRF expression subtypes and prognostic signatures adjusting for TNM stage or SSIGN score ……..………………………………………………………………27

Supplementary Table S5, related to Fig. 5. Summary of 5'-tRF and 3′-tRF expression subtypes and subtype-specific signatures across cancer types …………………………………………….27

Supplementary Table S6, related to Fig. 6. Tissue specificity of 11 cancer driver tRFs………27

Supplementary Table S7, related to Fig. 7 and “Supplementary Methods”. Information on antibody and primers used in this study………………………………………………………..27

Supplementary References …………………………………………………………28

Supplementary Methods

Subcellular localization of tRFs

Short RNA-Seq data of subcellular compartments (nucleus and cytoplasm) of A549 cell line in ENCODE project were obtained from GEO (GSE24565). The small RNA annotation, including miRNAs, small nuclear RNAs (snRNAs), small nucleolar RNAs (snoRNAs), small cytoplasmic RNAs (scRNAs) and Piwi-interacting RNAs (piRNAs), was downloaded from GENCODE (v19) (http://www.gencodegenes.org). Reads were aligned to the human hg19 UCSC genome annotation using BWA(1) and then these mapped reads were intersected with the small RNA annotation using bedtools. The total aligned reads within small RNA annotation were normalized by RPM. The overall reads mapped to 5′-tRFs, 3′-tRFs and 3′U-tRFs were called and normalized using our tRFs computational pipeline. From the normalized total expression, we calculated the relative fraction among different small RNA types in nucleus and cytoplasm and between nucleus and cytoplasm for each small RNA type.

Tissue specimens

A total of 10 pairs of non-small cell lung cancer (NSCLC), 9 pairs of stomach adenocarcinoma (STAD), 10 pairs of liver hepatocellular carcinoma (LIHC) and corresponding adjacent non-tumor tissues were obtained from patients who underwent surgery at Sir Run Run Shaw Hospital of Zhejiang University. A total of 10 pairs of uterine corpus endometrial carcinoma (UCEC) and corresponding adjacent non-tumor tissues were obtained from the Women's Hospital of Zhejiang University. A total of 10 pairs of kidney renal clear cell carcinoma (KIRC) and corresponding adjacent non-tumor tissues were from The First Affiliated Hospital of Zhejiang University. A total of 10 pairs of breast invasive carcinoma (BRCA) and corresponding adjacent non-tumor tissues were from The Second Affiliated Hospital of Zhejiang University. A total of 9 pairs of head and neck squamous carcinoma (HNSC) and adjacent non-tumor tissues were obtained from Shanghai Ninth People's Hospital of Shanghai Jiaotong University. The diagnosis of all tissues was confirmed histopathologically, and the TNM clinical stages were determined based on the American Joint Committee on Cancer and the Union for International Cancer Control in 2002. The study protocol was approved by the Institutional Review Board of these hospitals. All samples used in the current study were obtained at the time of diagnosis before any treatment was administered.

Jensen Shannon divergence (JS) cleavage score

The JS divergence is used to compare two discrete probability distributions, and is calculated as:

$$\boldsymbol{JS}(\boldsymbol{e^1}, \boldsymbol{e^2}) = \boldsymbol{H}\left(\frac{\boldsymbol{e}^1+\boldsymbol{e}^2}{\boldsymbol{2}}\right) - \frac{\boldsymbol{H}(\boldsymbol{e}^1)+\boldsymbol{H}(\boldsymbol{e}^2)}{\boldsymbol{2}} \qquad (1)$$

where $\boldsymbol{H}$ is the Shannon entropy of the distribution:

$$\boldsymbol{e^1} \text{ and } \boldsymbol{e^2} = (e_1, e_2, \ldots, e_n) \text{ where } 0 \le e_i \le 1 \text{ and } \sum_{i=1}^{n} e_i = 1$$

$$\boldsymbol{H}(\boldsymbol{e^1}) \text{ and } \boldsymbol{H}(\boldsymbol{e^2}) = -\sum_{i=1}^{n} e_i \log(e_i) \qquad (2)$$

To quantify the distribution pattern of cleavage among different cancer types (or tissues) and tRNA genes, we defined a JS cleavage score that was modified from the method of Calibi et al (2). For each cancer type, let x_{mn} be the reads per million mapped reads (RPM) value of reads ending in the m-th position relative to 5′ end or 3′ CCA end of a particular tRNA in the n-th sample, where m

ranges from 15 to 30 (namely, 15-30nt), and *n* ranges from 1 to N (N is the sample size for this cancer type). Then, the expression values were Log2-tranformed and denoted as $p_{mn} = \log_2(x_{mn} + 1)$. To obtain robust estimation of the expression, the expression levels were averaged across samples for each cancer type, $\bar{p}_m = \sum_{n=1}^{N} p_{mn}/N$. The distribution pattern of tRNA cleavage for a particular cancer type was defined as a vector:

$$\boldsymbol{C} = (c_{15}, \dots, c_m) = (\bar{p}_{15}/\sum_{i=15}^{m} \bar{p}_i, \dots, \bar{p}_m/\sum_{i=15}^{m} \bar{p}_i)$$

where m = 15 … 30; c_j is the relative abundance of fragments ended in the *j*-th position relative to 5′ end and 3′ CCA end of a particular mature tRNA.

Finally, the JS cleavage score between two cancer types, a and b, with tRNA cleavage profiles as $\boldsymbol{C^a}$ and $\boldsymbol{C^b}$, respectively, is defined as:

$$\boldsymbol{JS}_{cleavage\ score} = \mathbf{1} - \sqrt{\boldsymbol{JS}(\boldsymbol{C^a}, \boldsymbol{C^b})}$$

where JS cleavage score ranges from 0 to 1. A score of 0 indicates that the mature tRNAs have the similar cleavage pattern between two cancer types.

With the same method as described above, the JS cleavage score between two mature tRNAs is calculated below. First, each tRNA cleavage profile was averaged across different cancer types:

$$\boldsymbol{T} = (t_{15}, \dots, t_m) = (\sum_{k=1}^{K} c_{15,k}/K, \dots, \sum_{K=1}^{K} c_{m,k}/K)$$

where m = 15 … 30; k=1 … K; K is the number of cancer types;

Then, the JS cleavage score between two tRNAs, a and b, with tRNAs cleavage profiles as $\boldsymbol{T^a}$ and $\boldsymbol{T^b}$, respectively, is defined as:

$$\boldsymbol{JS}_{cleavage\ score} = \mathbf{1} - \sqrt{\boldsymbol{JS}(\boldsymbol{T^a}, \boldsymbol{T^b})}$$

where JS cleavage score ranges from 0 to 1. A score of 0 indicates that the two mature tRNAs have the similar cleavage profile.

Tissue specificity (TS) score

To assess tissue specific expression of tRFs, the method developed by Calibi et al. was used to define TS score (2). In brief, a vector showing the tRF expression profile was constructed:

$$\boldsymbol{E} = (e_1, \dots, e_J) = (\bar{s}_1/\sum_{i=1}^{J} \bar{s}_i, \dots, \bar{s}_J/\sum_{i=1}^{J} \bar{s}_i)$$

where *J* is the number of tissue types (or cancer types); $\bar{s}_i$ is the average tRF expression across samples for each tissue (or cancer type); e_j is the relative abundance of tRFs among different tissue types (or cancer types). The TS score for tissue type *t* is thus calculated as:

$$\boldsymbol{TS}_{score,t} = \mathbf{1} - \sqrt{\boldsymbol{JS}(\boldsymbol{E}, \boldsymbol{E^t})}$$

where $\boldsymbol{E^t}$ is a hypothesized expression profile showing tRFs expressed in only one tissue type (or cancer type). $\boldsymbol{E^t}$ can be listed as:

$$\boldsymbol{E^1} = (\mathbf{1}, \mathbf{0}, \dots, \mathbf{0}), \boldsymbol{E^2} = (\mathbf{0}, \mathbf{1}, \dots, \mathbf{0}), \dots, \boldsymbol{E^t} = (\mathbf{0}, \mathbf{0}, \dots, \mathbf{1}, \dots, \mathbf{0}), \dots, \boldsymbol{E^J} = (\mathbf{0}, \mathbf{0}, \dots, \mathbf{1})$$

where t = 1 … *J*.

The TS score of a particular tRF is defined as:

$$\boldsymbol{TS}_{score} = argmax(\boldsymbol{TS}_{score,t})$$

where t=1 … *J.* A score of 1 indicates that the tRF is expressed in only one tissue type (or cancer type).

Differential expression analysis

To compare the expression profile pattern of small non-coding RNA (snRNAs; e.g. tRFs and miRNAs) between tumor and normal samples, only the cancer types with more than 15 normal samples were retrieved, which resulted in 12 cancer types available. Differentially expressed snRNAs were defined as those with Benjamini-Hochberg corrected *P*-value < 0.05 (*P*-value was obtained using Wilcoxon rank sum test and permutation test) and fold change between tumor and normal samples more than 2 or less than 0.5.

Survival analysis in kidney cancer

Sequencing platform information of 544 kidney renal clear cell carcinoma (KIRC) samples was obtained from the ICGC Data Portal (https://dcc.icgc.org), including 283 specimens from Genome Analyzer IIx and 261 from HiSeq 2000. To achieve robust assessment, they were used as a discovery and validation set, respectively. The SSIGN scores of KIRC patients were calculated using a previously described method based on tumor stage, size, grade, and necrosis information retrieved from the ICGC Data Portal (https://dcc.icgc.org) (3). The SSIGN score of patients was divided into four groups, including low risk (0-2), low-medium risk (3-4), high-medium risk (5-6) and high risk (7-15). Kaplan-Meier curves were plotted to reveal survival differences among different tRF expression subtypes, the AJCC TNM stages and the SSIGN groups as well as tRF expression subtypes within each TNM stage and each SSIGN group. A log-rank test was used to evaluate statistical differences in survivals. In addition, two multivariate Cox proportional hazards model analyses adjusted for the AJCC TNM stage or the SSIGN score, respectively, as well as age, were performed to assess whether tRF expression subtypes were independent prognostic factors.

Identification of tumor subtypes based on tRF expression

For all 15 cancer types, non-negative matrix factorization (NMF) consensus clustering analysis was performed on the tRF expression profiles to identify tRF expression subtypes. The lowly variable tRFs with interquartile range, IQR<0.5, were filtered before the NMF cluster analysis. The optimal number of subtypes was determined from the cophenetic correlation coefficients. Additionally, representative tRF signatures were extracted for each subtype using a procedure previously developed by Kim and Park (4), which was implemented in NMF software. Those tRFs whose featuring scores were larger than 0.75 were defined as subtype-specific tRF signatures. Kaplan–Meier curves were plotted for these tRF expression subtypes; a log-rank test was used to evaluate their statistical differences in survivals between different cancer types. The number of subtypes and their subtype-specific signatures for each cancer type were presented in **Supplementary Table S5**.

Identification of supercluster

To detect a robust supercluster, 300 5′ tRFs and 120 3′ tRFs that were shared by at least 13 cancer types were selected for the analysis. First, *t*-test statistics were calculated for these tRFs through comparison between expression level of tRFs in each tRF expression subtype and that in other subtypes within the same cancer type. As a result, a *t*-test statistics matrix was obtained, which had rows and columns representing tRFs (300 5′-tRFs and 120 3′-tRFs) and subtypes (49 5′-tRFs-based

subtype and 52 3′-tRFs-based subtype), respectively. Then, an unsupervised hierarchical cluster analysis was performed on the *t*-test statistics matrix using ward linkage and Spearman correlation distance. Three superclusters were determined based on our prior knowledge, maximal correlation among subtypes, the least number of clusters and each cluster containing most cancer types.

Single sample gene set enrichment analysis (ssGSEA)

ssGSEA was performed to quantify the extent to which a gene set was activated or inactivated in each sample of a given data set. This was an extension of the commonly used GSEA package. The expression values of genes were first rank-normalized in a given sample. An ssGSEA score was obtained through comparing the difference between the empirical cumulative distribution function of genes in a gene set and that of the remaining genes. The detailed procedures can be found in a previous study (5). Then, the ssGSEA score was calculated for the 3,300 gene signatures/modules as well as proliferation, immune and stroma signatures in all samples across 15 cancer types in the study.

Pathway score using RPPA

Each of the analyzed pathways and their members were obtained from a previous study (6). Based on TCGA RPPA data, a pathway score was calculated for each sample across cancer types using the method described previously. Briefly, each protein expression level was normalized by *z*-score across all samples, and a pathway score $\boldsymbol{S}$ was defined as:

$$\boldsymbol{S} = \sum e^p - \sum e^n$$

where e^p was the normalized level of all positive regulatory proteins in a particular pathway and e^n was the normalized level of all negative regulatory proteins in this pathway.

Identification of cancer driver tRFs

The rich TCGA data resource provided us opportunities to establish an integrated strategy to mine potential cancer driver tRFs that potentially drive cancer phenotypes, mainly including two steps: 1) analysis in a single cancer type. For each of 12 cancer types with at least 15 normal samples, differentially expressed tRFs were identified using the method described above. Additionally, prognosis-associated tRFs were detected using the univariate Cox model (FDR < 0.05) for each of these cancer types. 2) analysis across cancer types. For each of differentially expressed tRFs, the *P* values from multiple cancer types were subsequently combined using Fisher's method, resulting in one integrated *P* value for each tRF. For each of prognosis-associated tRFs, one combined *P* value was estimated in the same way as above. Owing to tRFs being over-expressed in most cancer types (from our findings), we predominantly aimed to screen out candidate tRFs with oncogenic roles. Therefore, the cancer driver tRF candidate needed to meet the following conditions: 1) both integrated *P* values were < 0.05, and 2) upregulated in tumor tissues and high expression was associated with a worse prognosis.

GO analysis

Gene ontology (GO) analysis was performed using Database for Annotation, Visualization and Integrated Discovery (DAVID) (v6.8; https://david.ncifcrf.gov).

GSEA analysis

Gene set enrichment analysis (GSEA) was performed to evaluate whether cell-cycle related gene set revealed statistically significant differences between two different biological states (http://software.broadinstitute.org/gsea/index.jsp).

Dumbbell-PCR for tRFs and four-leaf clover qRT-PCR for tRNAs

Total RNA was extracted from tissues or cultured cells using TRIzol reagent (Invitrogen). The Dumbbell-PCR (Db-PCR) for tRF quantification was performed as described previously with modifications (7). Briefly, 1μg of tissues or cellular total RNA was incubated with 20pmol of the 3′or 5′-Db-adapter and 2μl 5× annealing buffer (Beyotime Biotechnology, China) in a 10μl reaction mixture at 95°C for 3min following by nature cooling for 2h. To ligate the annealed adapter to target tRFs, 10μl of the 1× reaction buffer containing 1U of Rnl2 (New England Biolabs) was added to the mixture. The entire mixture (20μl) was incubated at 37°C for 1h, followed by overnight incubation at 4°C. For reverse transcription, the ligated RNA (5μl) was mixed with gene specific primer using the PrimeScript™ RT reagent Kit (Takara). The resultant cDNA solution was added to the real-time PCR mixture containing 5μl of 2× Premix Ex Taq reaction solution (Takara), 100nM or 400nM TaqMan probe, and 2pmol each of the forward and reverse primers (10μl in total), and measured using the ViiATM7 Real-Time PCR System (Applied Biosystems).

The four-leaf clover qRT-PCR (FL-PCR) for mature tRNA quantification was performed as described previously with modifications (8). Total RNAs were incubated at 37°C for 40 min in 20 mM Tris-HCl (pH 9.0) to remove the amino acids from the mature tRNAs (deacylation treatment), followed by ethanol precipitation. Then the annealing and ligation of adapters to mature tRNAs and the TaqMan qRT-PCR for mature tRNAs were performed as Db-PCR. The sequences of adapters, primers and probes were presented in **Supplementary Table S7**.

Northern blot

Total RNA was purified using TRIzol reagent (Invitrogen) from A549 and Calu1 cell lines. Northern blot was performed with 80 ug of total RNA from each sample. For 5′-Ile-AAT-8-1-L20 detection, 100 pmol 5’-GCCGACTGAGCTAACCGGCC-3’ probe was labeled with digoxin on the 3’-end by DIG Oligonucleotide Tailing Kit (2nd Generation) (Roche) and the labeling efficiency was determined by DIG High Prime DNA Labeling and Detection Starter Kit II (Roche) according to the manufacturer’s instructions. Total RNA was mixed with formamide in equal proportion and denatured at 95°C for 10 min, and then mixed with 10×loading dye after 10 min ice bath. RNA samples were resolved by 17% denaturing polyacrylamide gel containing 7 M urea, and then transferred on positively charged nylon membrane (Roche) using a semidry transfer apparatus (BioRad). After pre-hybridization for 2 hours at 37°C, hybridization was performed in DIG Easy Hyb solution (Roche) containing 0.1 mg/ml ploy(A) and 10 pM labeling probe. After over-night hybridization at 37°C, membranes were washed twice in 2×SSC and 0.1% SDS for 5 min at room temperature and twice in 0.5×SSC and 0.1% SDS for 15 min at 65°C and in washing buffer (0.1M Maleic acid, 0.15M NaCl, pH7.5, 0.3% Tween 20) for 2 min at room temperature. After blocking for 30 min in blocking solution (Roche), membranes were incubated with Anti-Digoxigenin-AP Fab fragments (Roche) for 30 min at room temperature. Finally, membranes were washed twice in washing buffer for 15 min and balanced in detection buffer (0.1M Tris-HCl, 0.1M NaCl, pH9.5) for 5 min, then images were obtained by exposure to film.

RPPA protein data

Reverse phase protein lysate array (RPPA) profile data of the studied 15 cancer types in TCGA were downloaded from ICGC Data Portal (https://dcc.icgc.org) (**Supplementary Table S1**). The log-transformed values of each antibody were used for analysis.

miRNA-seq data

miRNA expression profile data of the studied 15 cancer types in TCGA were downloaded from the ICGC Data Portal (https://dcc.icgc.org) (**Supplementary Table S1**). The original read counts of miRNA were normalized by log_2-transformed of RPM using our tRFs computational pipeline. Additionally, low-expressed miRNAs that 90th quantile expression levels were smaller than 1 RPM were filtered and those remaining were used for subsequent analysis.

mRNA-seq data

mRNA expression profile data of the studied 15 cancer types in TCGA were downloaded from the ICGC Data Portal (https://dcc.icgc.org) (**Supplementary Table S1**). The expression values of protein-coding genes (PCGs) were normalized using the Read Per Kilobase per Million mapped reads (RPKM). Low-expressed PCGs with 90th quantile RPKM<1 were removed and the remaining log_2-transformed RPKM values were used for subsequent analysis.

Clinical data of TCGA patients

Clinical information of TCGA patients, including survival time, age, tumor stage and grade, was retrieved from the ICGC Data Portal (https://dcc.icgc.org) and was further processed for subsequent analyses.

Cell culture

A549 and Calu1 cells were obtained from ATCC and were cultured in RPMI-1640 medium (GIBCO-BRL) supplemented with 10% FBS and 1% antibiotic/antimycotic solution. Cells were incubated in a CO2 incubator (Thermo Fisher Scientific) maintained at 37°C with humidified air and 5% CO_2.

Exogenous tRF and siRNA transfection

A549 and Calu1 cells were transfected with synthesized 5′-IleAAT-8-1-L20 mimics, siRNA or antisense inhibitor using 6-well plates with GeneMuteTM reagent (**Supplementary Table S7**). After 8hr of incubation, transfection media were replaced with fresh media. Cells were subjected to in vitro and in vivo studies 24hr after transfection.

Cell proliferation assay

Cell proliferation was determined by incorporation of cell counting kit-8 (Dojindo Laboratories, Kumamoto, Japan). In brief, cells were transfected with 5′-IleAAT-8-1-L20 mimics or siRNAs using GeneMuteTM reagent (signaGenTM Laboratories). 24h after transfection, approximately 2,000 cells were placed into each well of 96-well plates. CCK-8 solution was added at 0h, 24h, 48h, 72h and 96h after placing. After adding 10ul CCK-8 solution, cells were incubated for 1.5h at 37°C. The absorbance was measured at 450nm.

Clone formation assay

Lung cancer cells were transfected with the siRNAs for 24h. 1500 cells were then plated in 6-well culture plates and cultured in RPMI 1640 medium supplemented with 10% FBS for one week. These cells were then fixed with methanol and stained with crystal violet solution.

Edu incorporation assay

Cell proliferation was assessed by Cell-Light EdU DNA cell proliferation kit (RiboBio, Guangzhou, China), according to the manufacturer's instructions.

Migration and invasion assay

In vitro migration and invasion assays were performed using Transwell chambers. Calu1 and A549 cells were transfected with siRNA or negative siRNA for 24h. Then these cells were cultured with serum-free RPMI 1640 for 24h. These cells were then detached and resuspended in RPMI 1640 medium with serum-free. 3×10^4 cells in 300ul cell suspensions were added in the upper transwell chambers for migration assay or the upper transwell chambers coated with Matrigel for invasion assay, and the RPMI 1640 with 10% FBS was added to the bottom chamber. Migrated and invasive cells were stained with 0.1% crystal violet. Images were captured from each membrane and the number of migratory cells was counted under a microscope.

Cell Cycle assay

Cell cycle assay was used to detect whether 5′-IleAAT-8-1-L20 regulated the cell cycle. In brief, cells transfected with siRNA for 24h were then cultured with serum-free RIPM 1640 medium for 24h. Next, cells were cultured with serum medium for another18h. Cells were collected, followed by fixing with ice-cold 70% ethanol for 20min and stained with propidium iodide (PI, BD, San Diego, CA, USA). The cell cycle was determined by flow cytometry (FACS Calibur, BD, San Diego, CA, USA).

Western blotting

Cells were suspended in lysis buffer (50 mM Tris-HCl PH 8.0, 1% SDS, 1 mM EDTA, 5 mM DTT, 10 mM PMSF, 1 mM NaF, 1 mM Na3VO4, and protease inhibitor cocktail), and then denatured in boiling water for 10min. The cellular lysates were centrifuged at 13 000 rpm for 30 min. The protein concentration was determined by BCA assay (Thermo Fisher Scientific, Waltham, MA, USA). Equal amounts of proteins (40ug) were separated on 10% SDS-PAGE, then transferred onto a polyvinylidene fluoride (PVDF) membrane, blocked with 5% skim milk and immunoblotted with antibodies against pRB, RB, Cyclin A2, Cyclin B1, Cyclin D1, Cyclin E1, p27, p21 and GAPDH. Immunoreactive bands were developed by enhanced chemiluminescence reaction (Pierce) following standard protocols (**Supplementary Table S7**).

Immunoprecipitation experiment (RIP) assay

A total of $\sim2\times10^7$ A549 cells were incubated with 5μg anti-pan-AGO antibody (Millipore) or control IgG overnight with rotation at 4°C. The whole process was performed as the manufacturer described using Magna RIPTM RNA-Binding Protein Immunoprecipitation Kit (Millipore) (**Supplementary Table S6**).

Luciferase reporter assay

5'-IleAAT-8-1-L20 sequence was inserted into psiCHECK2 vector. Then, the constructed reporter vector was transfected into A549 cells with siControl or 5'-IleAAT-8-1-L20 siRNA. After 36 h culture, the cells were lysed with passive lysis buffer (Promega, Cat# E1910), and the reporter gene expression was assessed using a Dual Luciferase reporter assay system (Promega, Cat# E1910). Finally, firefly luciferase values were first normalized to a Renilla luciferase control and then normalized by reporter co-transfected with empty vector, and the efficiency of 5'-IleAAT-8-1-L20 knockdown was calculated. All transfection assays were carried out in triplicate.

Animal experiments

To further assess cell proliferation, 2×10^6 A549 cells treated with siCrtl and siRNA of 5′-IleAAT-8-1-L20 were injected subcutaneously into the left and right flank of immune-deficient mice (BALB/c Nude) with 4-week old age. Given that siRNAs are stable for a couple of days, when performing animal experiments, we injected siRNA or vehicle every four days into the tumor. Tumor growth was monitored with caliper measurements and tumor volumes were determined according to the formula: $Width^2 \times Length/2$. Four weeks later, primary tumor masses were explanted and fixed in 4% paraformaldehyde, and embedded in paraffin. Sections (5μm thick) were prepared for histopathologic examination. All experiments were performed in accordance with the Institutional Animal Care and Use Committee (IACUC) of the Zhejiang University, Hangzhou, China.

All information on antibody and primers used in this study was presented in **Supplementary Table S7**.

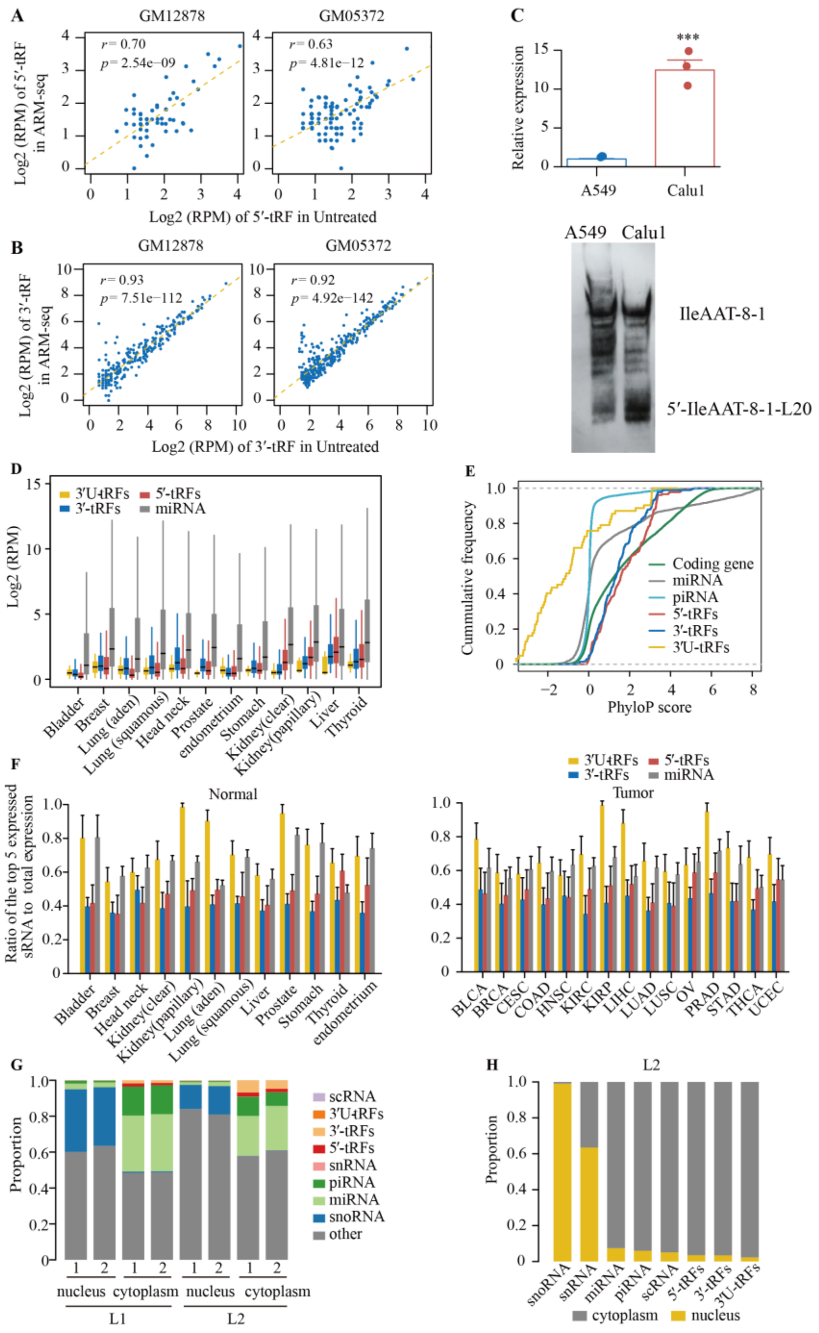

Supplementary Fig. S1, related to Fig. 1. Identification and quantification of endogenous tRFs across 15 cancer types. Comparison of 5′-tRFs (**A**) and 3′-tRFs (**B**) quantification using smRNA-seq and ARM-seq methods. Human B lymphocyte-derived cell lines GM05372 and GM12878 were used. Sequencing libraries of AlkB-treated and untreated RNA were prepared with NEBNext Small RNA Library Prep Kit for Illumina. (**C**) Db-PCR (*upper* panel) and northern blot (*lower* panel) for detection of tRF 5′-Ile-AAT-8-1-L20 derived from tRNA Ile-AAT-8-1 in A549 and Calu1 cell lines. (**D**) Distribution of tRF and miRNA expression across 12 normal tissues. (**E**) Conservation analysis of tRF compared with exon, miRNA and piRNA. Shown were cumulative frequency curves of evolutionary conservation scores of 5′-tRF, 3′-tRF, 3′U-tRF, exon, miRNA and piRNA sequence bases across 46 mammalian species. The x axis is PhyloP score and the larger score represents higher conservation. (**F**) The fraction of the top five expressed small ncRNAs (e.g. 5′-tRFs, 3′-tRFs, 3′U-tRFs and miRNAs) accounting for total expression levels of small ncRNAs across tumors and normal tissues. The

error bars indicate SD across samples. (**G**) The proportion of each annotated small ncRNA class in the nucleus and cytoplasm of A549 cells. 1 and 2 represent each of two replicates, respectively; L1, the library with only Tobacco Acid Pyro-phosphatase (TAP) prior to cloning; L2, the library with no prior treatment. (**H**) Comparison of the relative abundance of nucleus versus cytoplasm in ENCODE A549 cells across different small ncRNA classes for L2 library. The total reads of each small ncRNA class were normalized by RPM and then their relative abundance between the nuclear and cytoplasmic fraction was calculated.

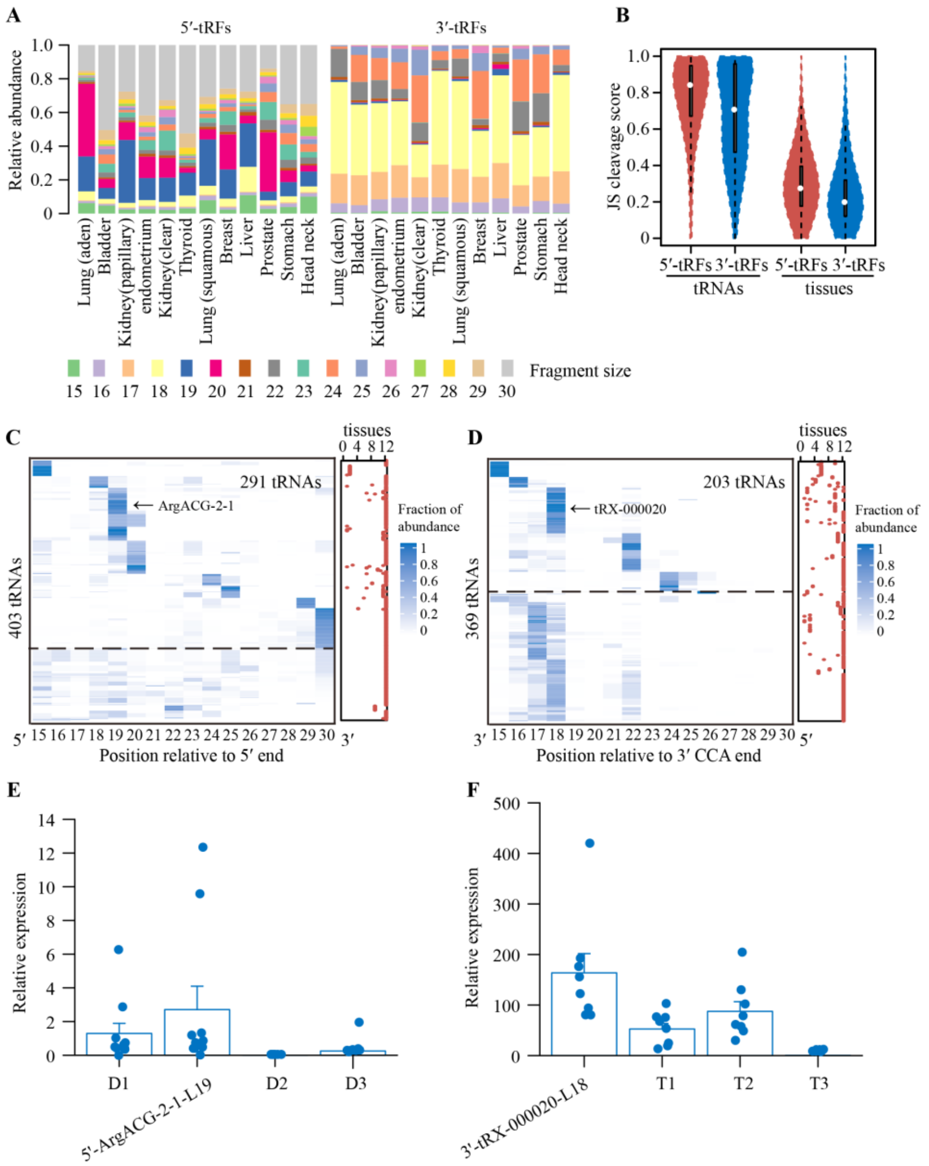

Supplementary Fig. S2, related to Fig. 2. tRFs resulted from the specific cleavage of tRNAs in cancer. (**A**) Relative abundance of different fragment sizes (15-30 nt) of 5′-tRFs (*left* panel) and 3′-tRFs (*right* panel) across normal tissues. Shown was the average relative expression value for each fragment size across samples. (**B**) Distribution of JS cleavage score calculated across different tRNAs or normal tissues. The Jensen-Shannon (JS) cleavage score used JS divergence (ranging from 0 to 1) as metric and JS=0 represents the same cleavage pattern among tRNAs or tissue types (see **"Supplementary Methods"** for details). (**C, D**) Cleavage profiles of 5′ (**C**) and 3′ (**D**) end of tRNAs that could generate 5′-tRFs and 3′-tRFs, respectively. Color intensity signifies relative ratio between reads mapped to a given position of tRNAs and total reads mapped to 5′ or 3′ end of tRNAs. Red dots in the *right* panel represent the number of normal tissue types harboring a similar cleavage pattern. Above the dash line indicates highly specific cleavage of 5′ and 3′ end tRNAs. (**E, F**) Db-PCR analyses of fragments derived from the major and other three minor cleavage positions of the two above described tRNAs, tRNA ArgACG-2-1 (**E**) and tRNA tRX-000020 (**F**), respectively. Prior to Db-PCR analysis, total RNA extracted from tumor specimens were pretreated with an rtStar^{TM} tRF&tiRNA Pretreatment Kit (Arraystar, USA), which removed 3'-aminoacyl and 3'-cP for 3' adaptor ligation, phosphorylated 5'-OH for 5'-adaptor ligation, and demethylated m1A, m1G, and m3C. D1, D2 and D3 represent 5′ end fragments; T1, T2, and T3 represent 3′ end fragments. The error bars indicate SD across samples (n=10 for 5′-tRFs; n=8 for 3′-tRFs).

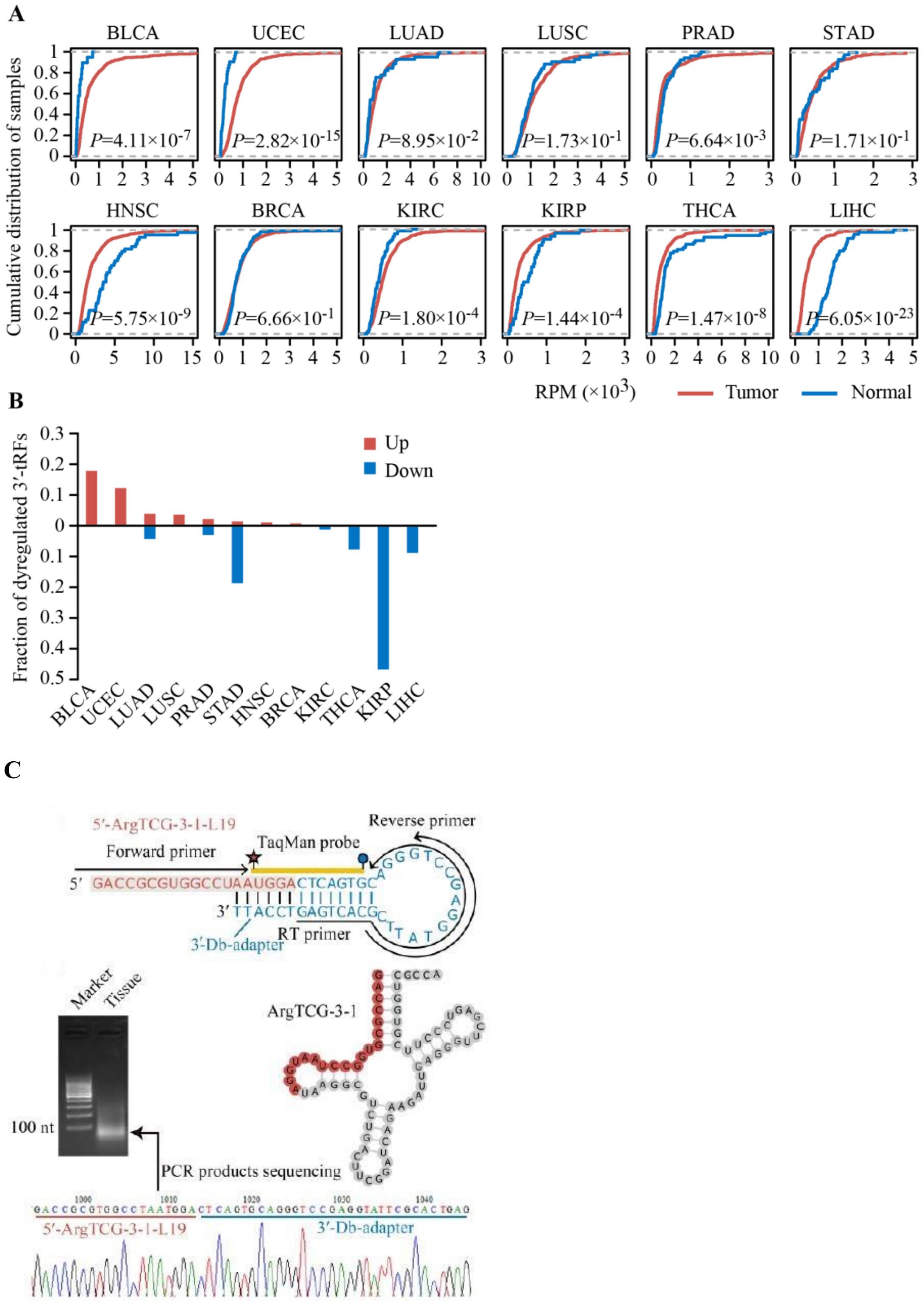

Supplementary Fig. S3, related to Fig. 3. The dysregulated expression of tRFs in Cancer.
(**A**) Empirical cumulative distribution plot of the overall 3′-tRF expression levels in tumor and normal specimens across 12 cancer types. RPM: reads per million mapped reads; P: p values. (**B**) The proportion of up-regulated and down-regulated 3′-tRFs across different cancer types. (**C**) Validation of 5′-ArgTCG-3-1-L19 by Db-PCR. The PCR products were then sequenced..

A

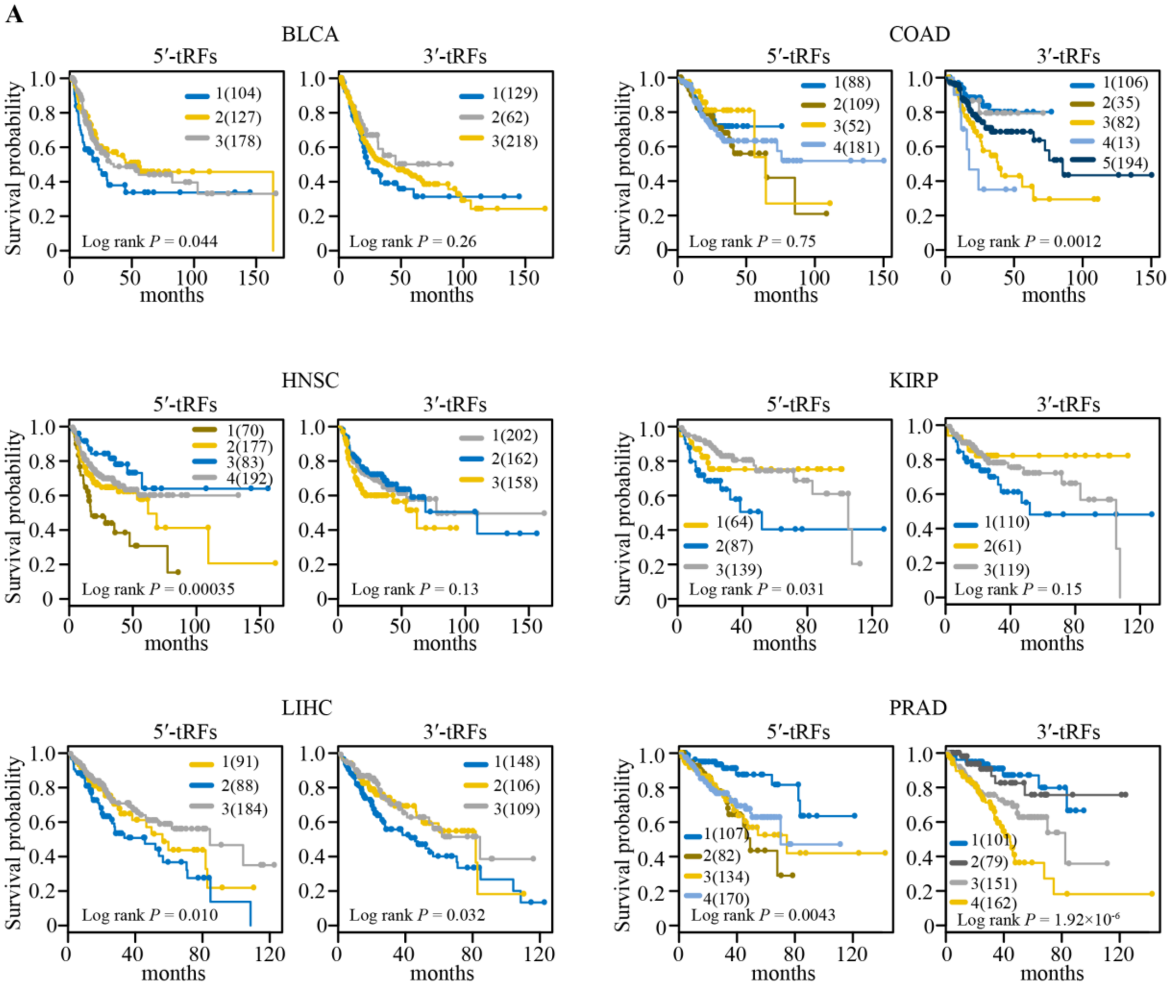

BLCA
5′-tRFs
1(104)
2(127)
3(178)
Log rank P = 0.044
3′-tRFs
1(129)
2(62)
3(218)
Log rank P = 0.26
COAD
5′-tRFs
1(88)
2(109)
3(52)
4(181)
Log rank P = 0.75
3′-tRFs
1(106)
2(35)
3(82)
4(13)
5(194)
Log rank P = 0.0012
HNSC
5′-tRFs
1(70)
2(177)
3(83)
4(192)
Log rank P = 0.00035
3′-tRFs
1(202)
2(162)
3(158)
Log rank P = 0.13
KIRP
5′-tRFs
1(64)
2(87)
3(139)
Log rank P = 0.031
3′-tRFs
1(110)
2(61)
3(119)
Log rank P = 0.15
LIHC
5′-tRFs
1(91)
2(88)
3(184)
Log rank P = 0.010
3′-tRFs
1(148)
2(106)
3(109)
Log rank P = 0.032
PRAD
5′-tRFs
1(107)
2(82)
3(134)
4(170)
Log rank P = 0.0043
3′-tRFs
1(101)
2(79)
3(151)
4(162)
Log rank P = 1.92×10⁻⁶
Survival probability
months

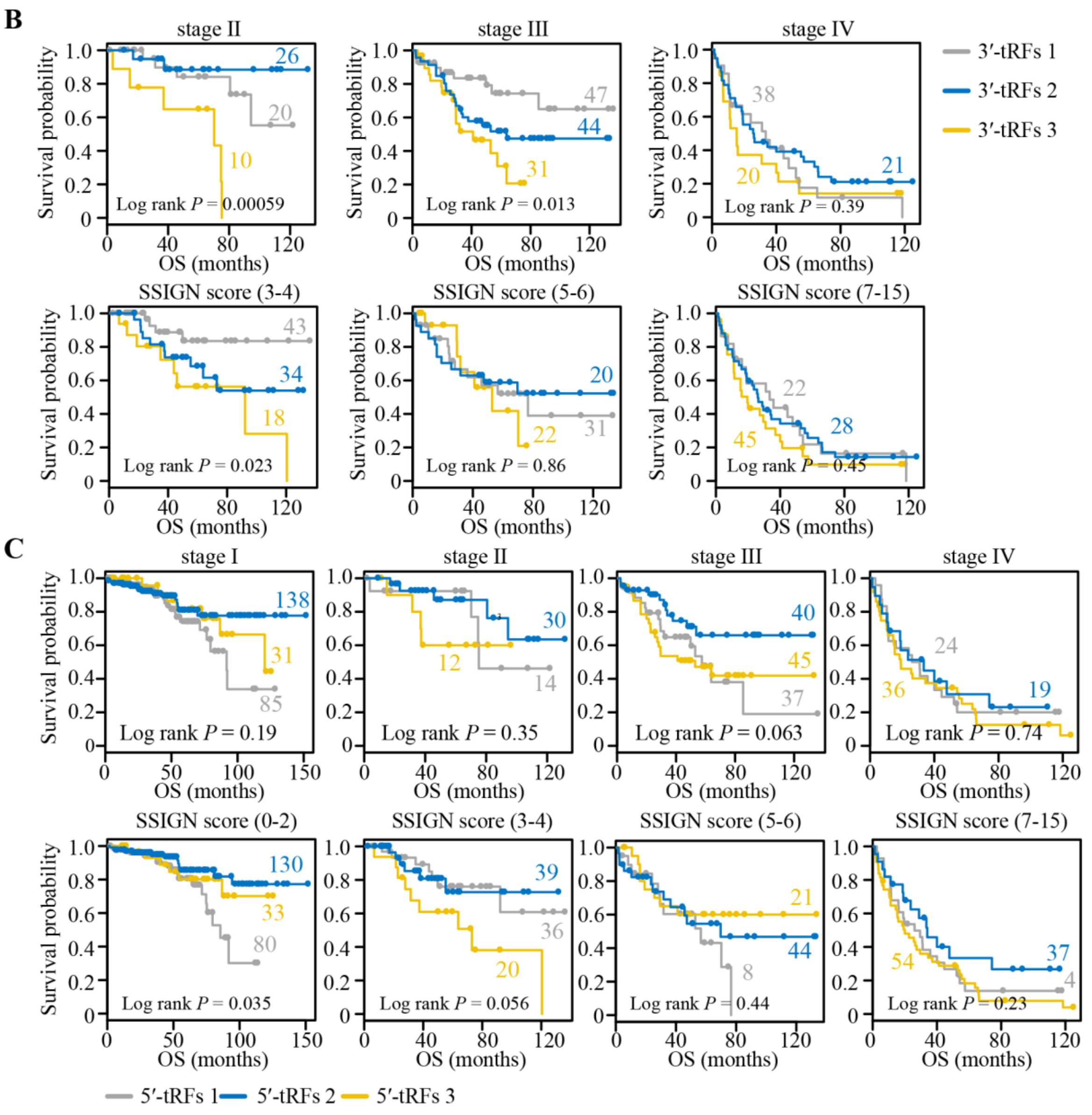

Supplementary Fig. S4, related to Fig. 4. tRF expression data reveal KIRC subtypes and improve outcome prediction. (**A**) 5′-tRF and 3′-tRF expression subtypes revealed distinct prognostic stratifications in several other cancer types. (**B**) Kaplan-Meier plot of the three 3′-tRFs subgroups in kidney renal clear cell carcinoma (KIRC) patients with TNM stage II, TNM stage III and TNM stage IV (*top* panel), respectively and in patients with low-medium risk SSIGN group, high-medium risk SSIGN group and high risk SSIGN group (*bottom* panel), respectively. (**C**) Kaplan-Meier plot of the three 5′-tRFs subgroups in the four TNM stages of KIRC patients (*top* panel), respectively and in the four SSIGN groups of patients (*bottom* panel), respectively.

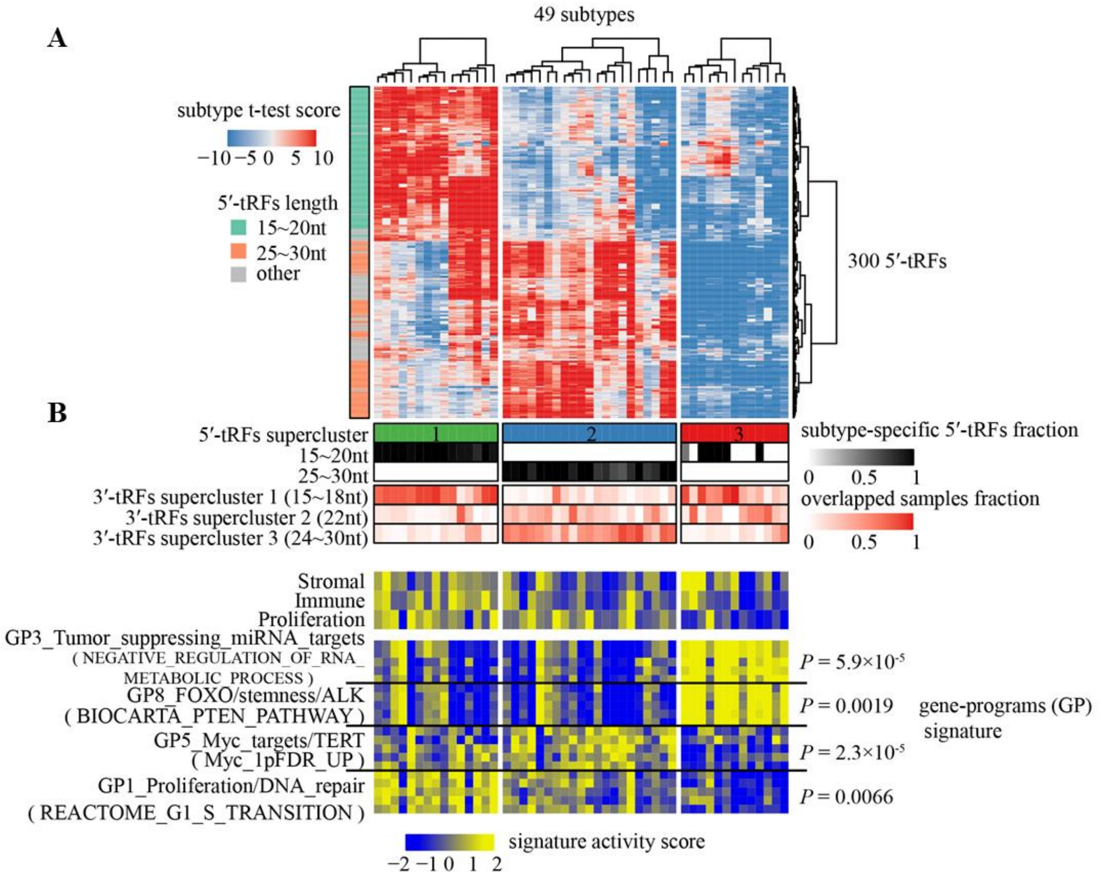

Supplementary Fig. S5, related to Fig. 5. Identification of biologically distinct supercluster via cluster analysis of 5′-tRF expression subtypes across 15 cancer types. (**A**) Unsupervised hierarchical clustering of 49 5′-tRF pan-cancer subtypes (see **Supplementary Table S5**) identified three superclusters highly correlated with the length of 5′-tRFs. Heatmap represented *t*-test score comparing the 5′-tRF expression level of each 5′-tRF expression subtype with that of other subclasses within the same cancer type. Below the heatmap (top to bottom): group identification numbers of supercluster; the fraction of subtype-specific 5′-tRF signatures belonging to a given group defined by length of 5′-tRFs (see **Supplementary Table S5**); overlapped fraction between samples of 5′-tRF supercluster and 3′-tRF supercluster. Left side bar shows the length of 5′-tRFs. (**B**) Biologically distinct characterization among three 5′-tRF superclusters. Heatmap reflected *t*-test statistic comparing the single sample GSEA (ssGSEA) score for gene/protein signatures of each 5′-tRF expression subtype with that of other subgroups within the same cancer type. Below the heatmap (top to bottom): the stroma, immune and proliferation signatures (*top* panel); 3,300 bimodal gene signatures (*middle* panel), grouped into 22 non-redundant *gene programs* (GP) symbolizing most cancer hallmarks. The most correlated *GP* with each 5′-tRF supercluster was selected according to the enrichment analysis (Fisher's Exact Test).

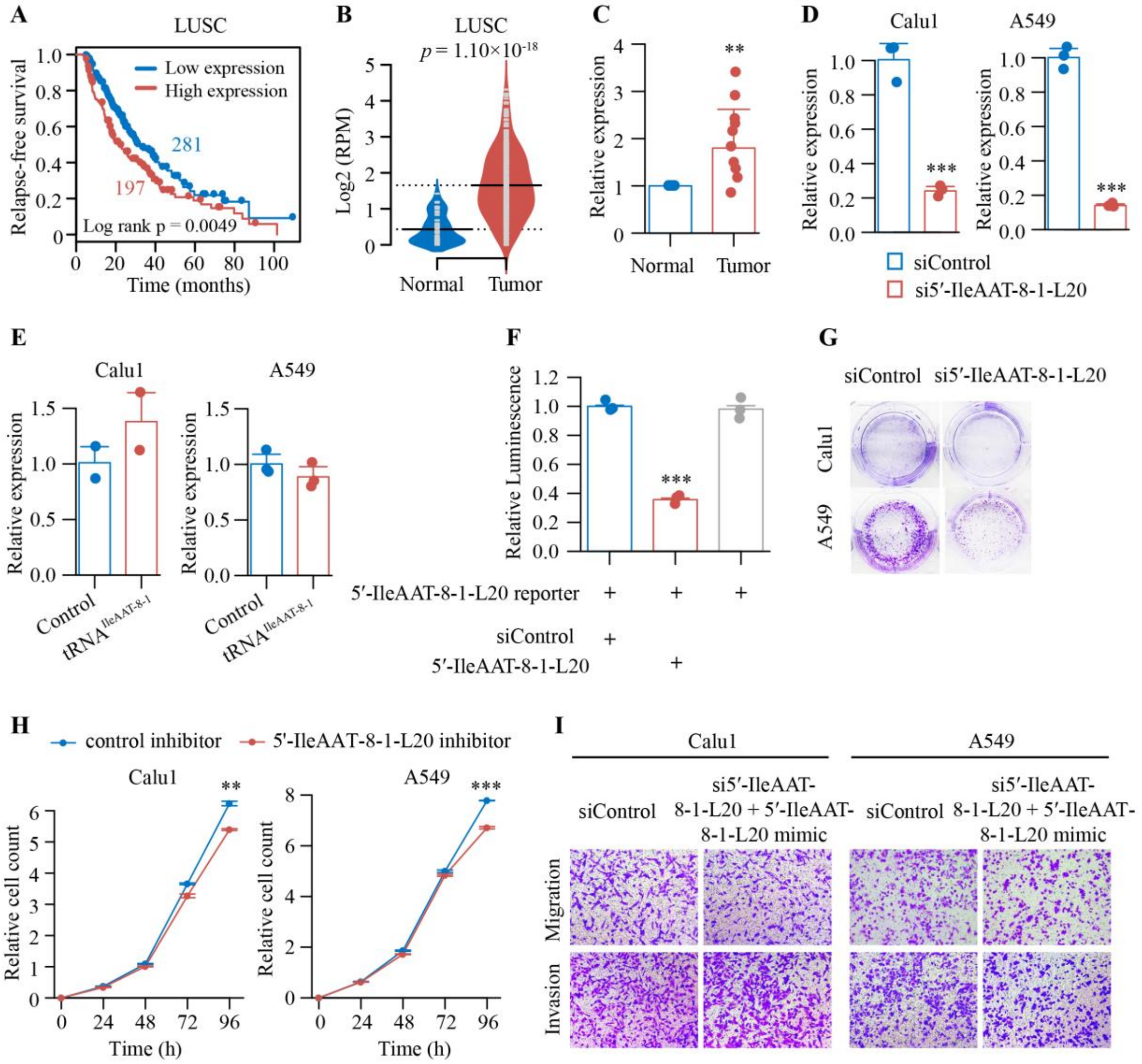
A
LUSC
Relapse-free survival
Low expression
High expression
281
197
Log rank p = 0.0049
Time (months)
B
LUSC
p = 1.10×10-18
Log2 (RPM)
Normal
Tumor
C
Relative expression
Normal
Tumor
D
Calu1
A549
Relative expression
siControl
si5′-IleAAT-8-1-L20
E
Calu1
A549
Relative expression
Control
tRNAIleAAT-8-1
F
Relative Luminescence
5′-IleAAT-8-1-L20 reporter
siControl
5′-IleAAT-8-1-L20
G
siControl
si5′-IleAAT-8-1-L20
Calu1
A549
H
control inhibitor
5'-IleAAT-8-1-L20 inhibitor
Calu1
A549
Relative cell count
Time (h)
I
Calu1
A549
siControl
si5′-IleAAT-8-1-L20 + 5′-IleAAT-8-1-L20 mimic
Migration
Invasion

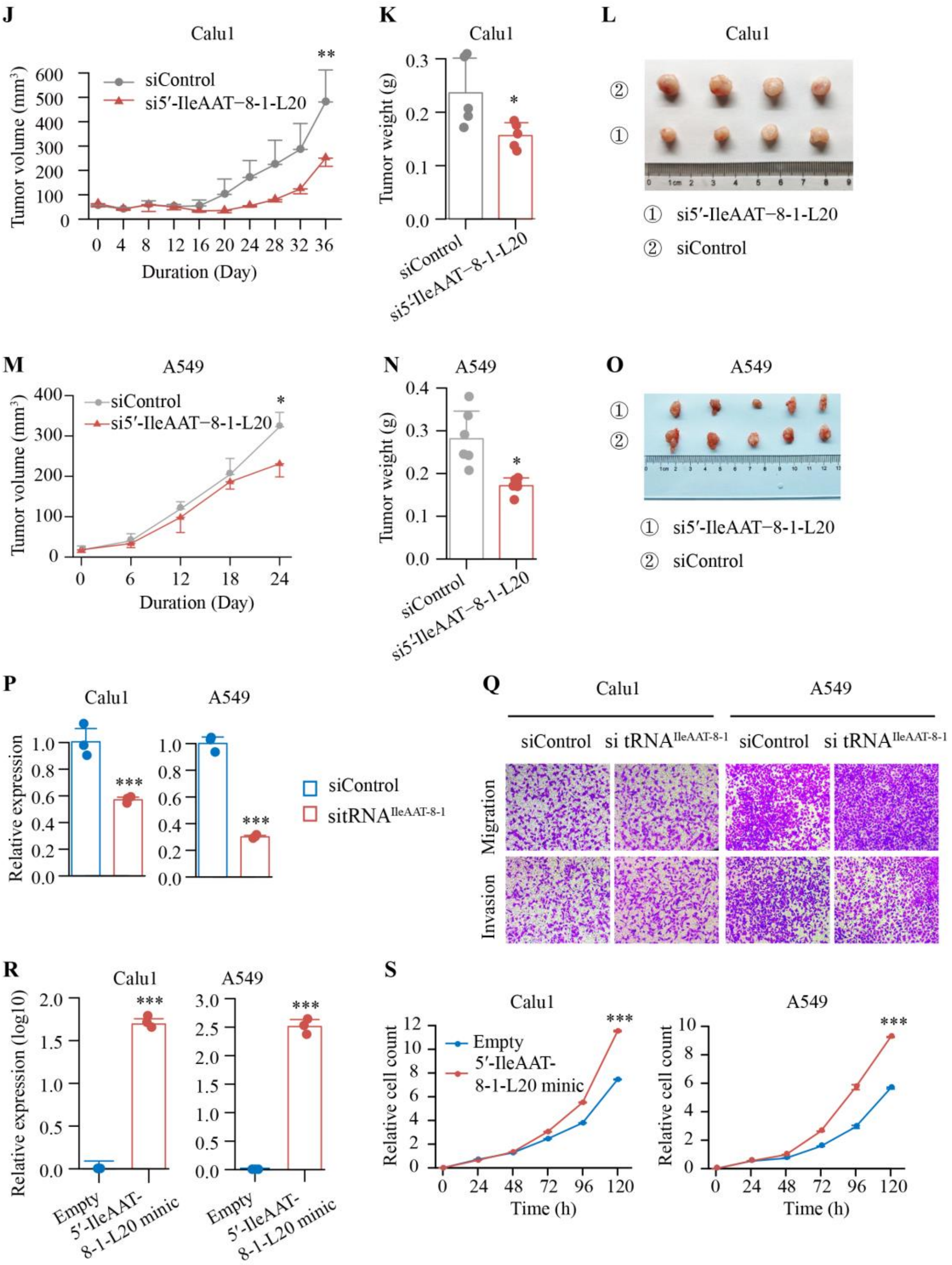

Supplementary Fig. S6, related to Fig. 6. Discovery of cancer driver tRFs using an effective approach for accurately exploring cross-cancer and platform trends. (**A**) Kaplan-Meier relapse-free survival analysis of patients grouped by the expression values of 5′-IleAAT-8-1-L20 in the TCGA Lung Squamous Cell Carcinoma (LUSC) cohort. *P* value is determined by log-rank test. (**B**) The expression level of 5′-IleAAT-8-1-L20 in Lung Squamous Cell Carcinoma (LUSC) (n = 478) and normal lung tissues (n = 45) from TCGA project. (**C**) Db-PCR analyses of 5′-IleAAT-8-1-L20 expression level in 10 lung tumors and matched normal tissues from our non-small cell lung cancer patients. (**D**) Relative expression change of 5′-IleAAT-8-1-L20 in Calu1 and A549 cells transfected with 5′-IleAAT-8-1-L20 siRNA or control siRNA. (**E**) Relative expression change of tRNAIleAAT-8-1 by FL-PCR in Calu1 and A549 cells transfected with 5′-IleAAT-8-1-L20 siRNA or control siRNA. tRNAIleAAT-8-1 is the precursor of 5′-IleAAT-8-1-L20. (**F**) Luciferase reporter assays showing the efficiency of siRNA targeted to 5′-IleAAT-8-1-L20. Firefly luciferase values were first normalized to a Renilla

luciferase control and then normalized by reporter co-transfected with empty vector. (**G**) Colony formation assay in Ca1u1 and A549 cells transfected with 5′-IleAAT-8-1-L20 siRNA or control siRNA. (**H**) Growth curves of Calu1 and A549 cells transfected with control inhibitor or 5′-IleAAT-8-1-L20 inhibitor. (**I**) Migration and invasion assays following knockdown of 5′-IleAAT-8-1-L20 and then transfection with 5′-IleAAT-8-1-L20 mimic in Calu1 and A549 cells. (**J, K, L**) Tumor volumes and weight of mouse xenografts subcutaneously injected with Caul cells expressing control siRNA or 5′-IleAAT-8-1-L20 siRNA, n = 5. (**M, N, O**) Tumor volumes and weight of mouse xenografts subcutaneously injected with A549 cells expressing control siRNA or 5′-IleAAT-8-1-L20 siRNA, n = 6. (**P**) Relative expression change of $tRNA^{IleAAT-8-1}$ in Calu1 and A549 cells transfected with $tRNA^{IleAAT-8-1}$ siRNA or control siRNA. (**Q**) Migration and invasion assays following knockdown of $tRNA^{IleAAT-8-1}$ in Calu1 and A549 cells. (**R**) Relative expression change of 5′-IleAAT-8-1-L20 in Calu1 and A549 cells transfected with empty or 5′-IleAAT-8-1-L20 overexpression vectors. (**S**) Growth curves of Calu1 and A549 cells transfected with empty or 5′-IleAAT-8-1-L20 overexpression vectors. The error bars indicate SD. *P <0.05, **P <0.01, ***P <0.001 using a two-sided Student’s t test.

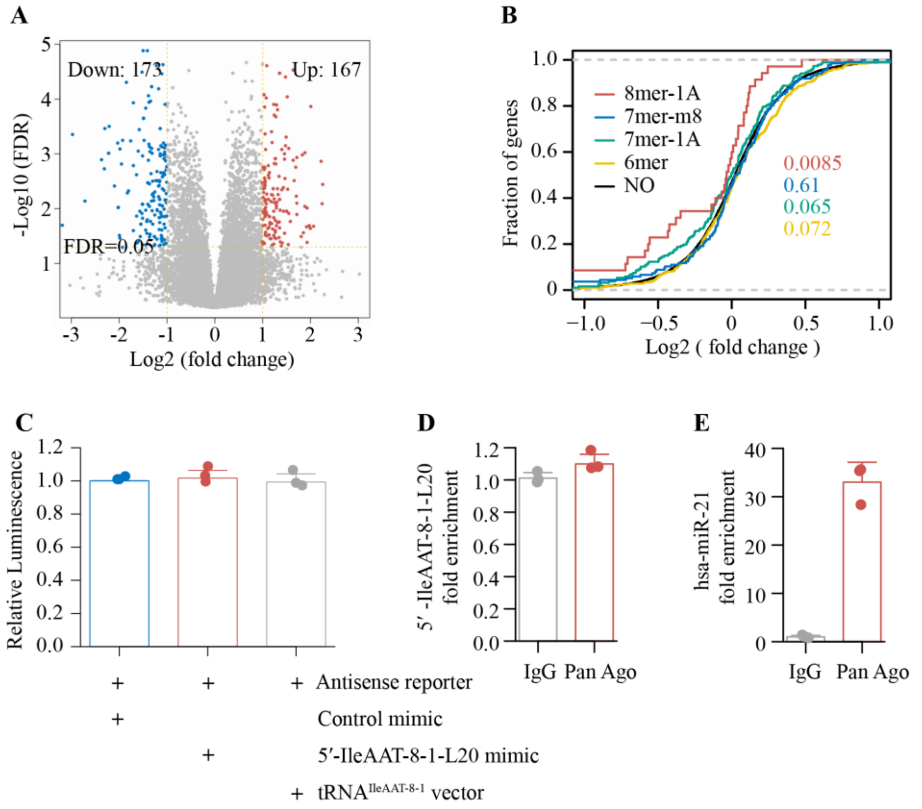

Supplementary Fig. S7, related to Fig. 7. 5′-IleAAT-8-1-L20 regulates the cell cycle in a non-miRNA manner. (**A**) Knockdown of 5′-IleAAT-8-1-L20 induced global gene expression changes in Calu1 and A549 cells. (**B**) Cumulative frequency curves for relative expression change of 5′-IleAAT-8-1-L20 targets and the remaining genes after knockdown of 5′-IleAAT-8-1-L20 in both Calu1 and A549 cell lines. 5′-IleAAT-8-1-L20 targets are predicted by TargetScan in a similar way to miRNA target prediction. If 5′-IleAAT-8-1-L20 functions as a miRNA, its targets will significantly shift toward to the right side relative to the non-targets. Four types of target sites commonly used in miRNAs were investigated for 5′-IleAAT-8-1-L20, including 8mer-1A (Watson–Crick match to miRNA positions 2–8 with an A opposite position 1), 7mer-m8 (position 2–8 match), 7mer-1A (position 2–7 match with an A opposite position 1) and 6mer (position 2–7 match).
(**C**) Relative expression level of antisense reporter in response to 5′-IleAAT-8-1-L20. Firefly luciferase values were first normalized to a Renilla luciferase control and then normalized by reporter co-transfected with empty vector. (**D**) 5′-IleAAT-8-1-L20 is not bounded by human Argonaute proteins. Db-PCR analyses of 5′-IleAAT-8-1-L20 in Pan-Ago and IgG control immunoprecipitation (IP) fractions of A549, respectively. (**E**) Db-PCR analyses of has-miR-21 in Pan-Ago and IgG control immunoprecipitation (IP) fractions of A549, respectively, used as a positive control. The error bars indicate SD of three independent experiments.

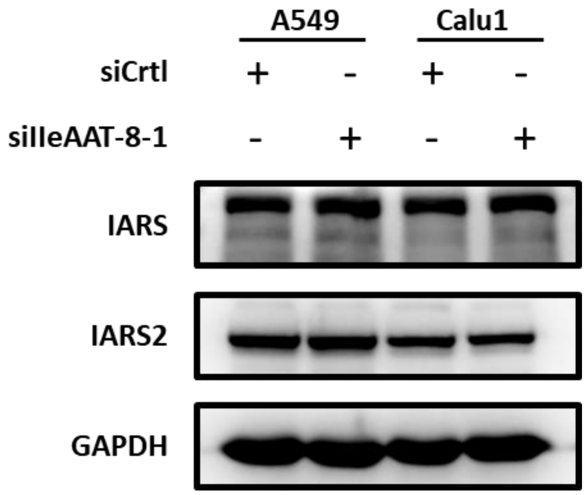

Supplementary Fig. S8 Effect of knockdown of IIeAAT-8-1 on IIeRS and IIeRS2 protein expression.

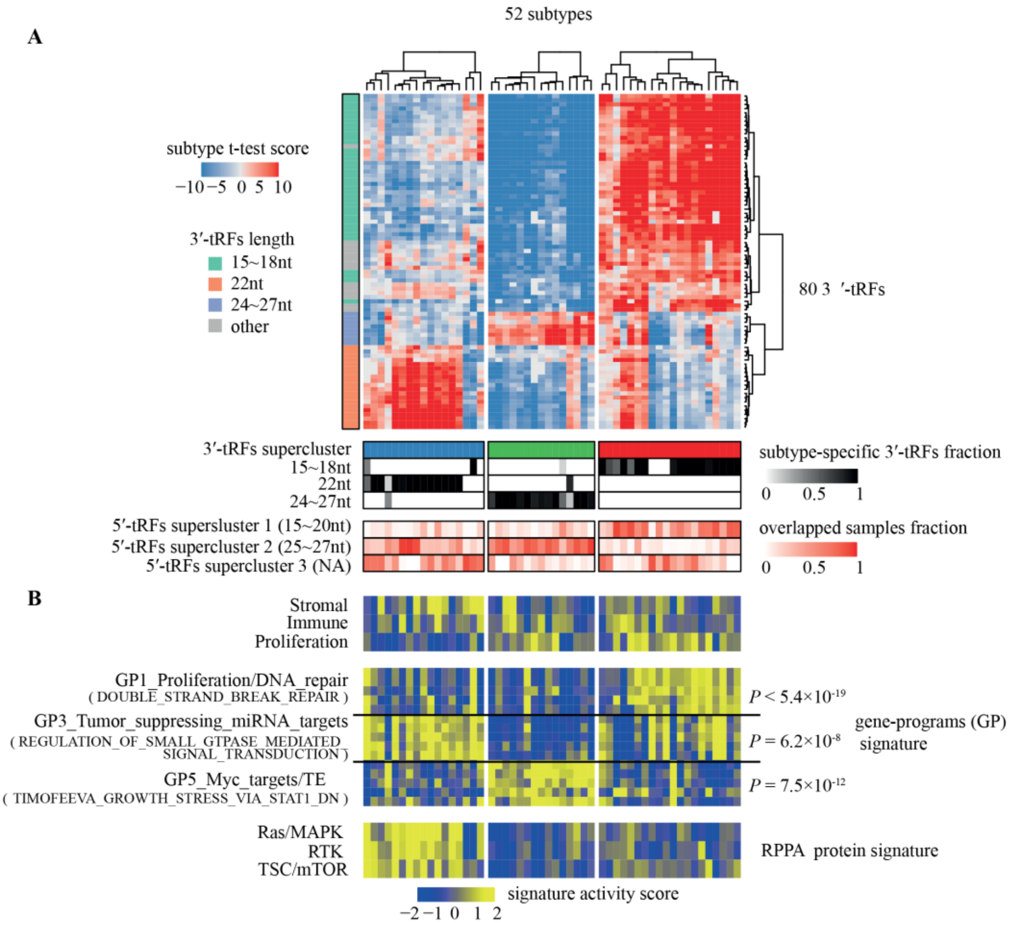

Supplementary Fig. S9. Identification of biologically distinct supercluster via cluster analysis of 3′-tRF expression subtypes (after removing ambiguous tRFs and tRFs with >27nt) across 15 cancer types. (**A**) Unsupervised hierarchical clustering of 52 3′-tRF pan-cancer subtypes identified three superclusters highly correlated with length of 3′-tRFs. Heatmap represented *t*-test score comparing the 3′-tRF expression level of each 3′-tRF expression subtype with that of other subgroups within the same cancer type. Below the heatmap (top to bottom): group identification numbers of supercluster; the fraction of subtype-specific 3′-tRF signatures belonging to a given group defined by length of 3′-tRFs (see **Supplementary Table S5**); overlapped fraction between samples of 5′-tRF supercluster and 3′-tRF supercluster. Left side bar shows the length of 3′-tRFs. (**B**) Biologically distinct characterization among three 3′-tRF superclusters. Heatmap reflected *t*-test statistic comparing the single sample GSEA (ssGSEA) score for gene/protein signatures of each 3′-tRF expression subtype with that of other subgroups within the same cancer type. Below the heatmap (top to bottom): the stroma, immune and proliferation signatures (*top* panel); 3300 bimodal gene signatures (*middle* panel), grouped into 22 non-redundant *gene programs* (GP) symbolizing most cancer hallmarks; RPPA protein signatures (*bottom* panel). The most correlated GP with each 3′-tRF supercluster was selected according to the enrichment analysis (Fisher's Exact Test; see **Supplementary Methods** for details).

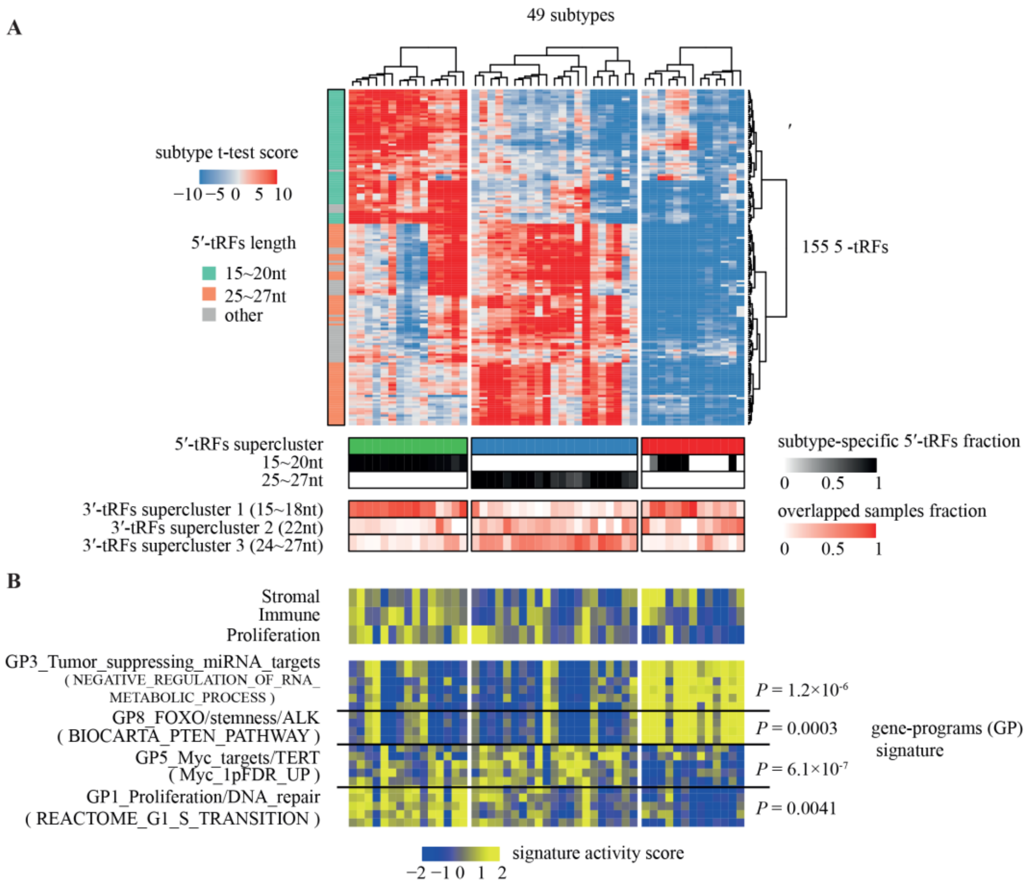

Supplementary Fig. S10. Identification of biologically distinct supercluster via cluster analysis of 5′-tRF expression subtypes (after removing ambiguous tRFs and tRFs with >27nt) across 15 cancer types. (A) Unsupervised hierarchical clustering of 49 5′-tRF pan-cancer subtypes (see Table S6 in the manuscript) identified three superclusters highly correlated with length of 5′-tRFs. Heatmap represented t- test score comparing the 5′-tRF expression level of each 5′-tRF expression subtype with that of other subclasses within the same cancer type. Below the heatmap (top to bottom): group identification numbers of supercluster; the fraction of subtype-specific 5′-tRF signatures belonging to a given group defined by length of 5′-tRFs (see **Supplementary Table S5**); overlapped fraction between samples of 5′-tRF supercluster and 3′-tRF supercluster. Left side bar shows the length of 5′-tRFs. (B) Biologically distinct characterization among three 5′-tRF superclusters. Heatmap reflected t-test statistic comparing the single sample GSEA (ssGSEA) score for gene/protein signatures of each 5′-tRF expression subtype with that of other subgroups within the same cancer type. Below the heatmap (top to bottom): the stroma, immune and proliferation signatures (top panel); 3,300 bimodal gene signatures (middle panel), grouped into 22 non- redundant gene programs (GP) symbolizing most cancer hallmarks. The most correlated GP with each 5′-tRF supercluster was selected according to the enrichment analysis (Fisher's Exact Test; see **Supplementary Methods** for details).

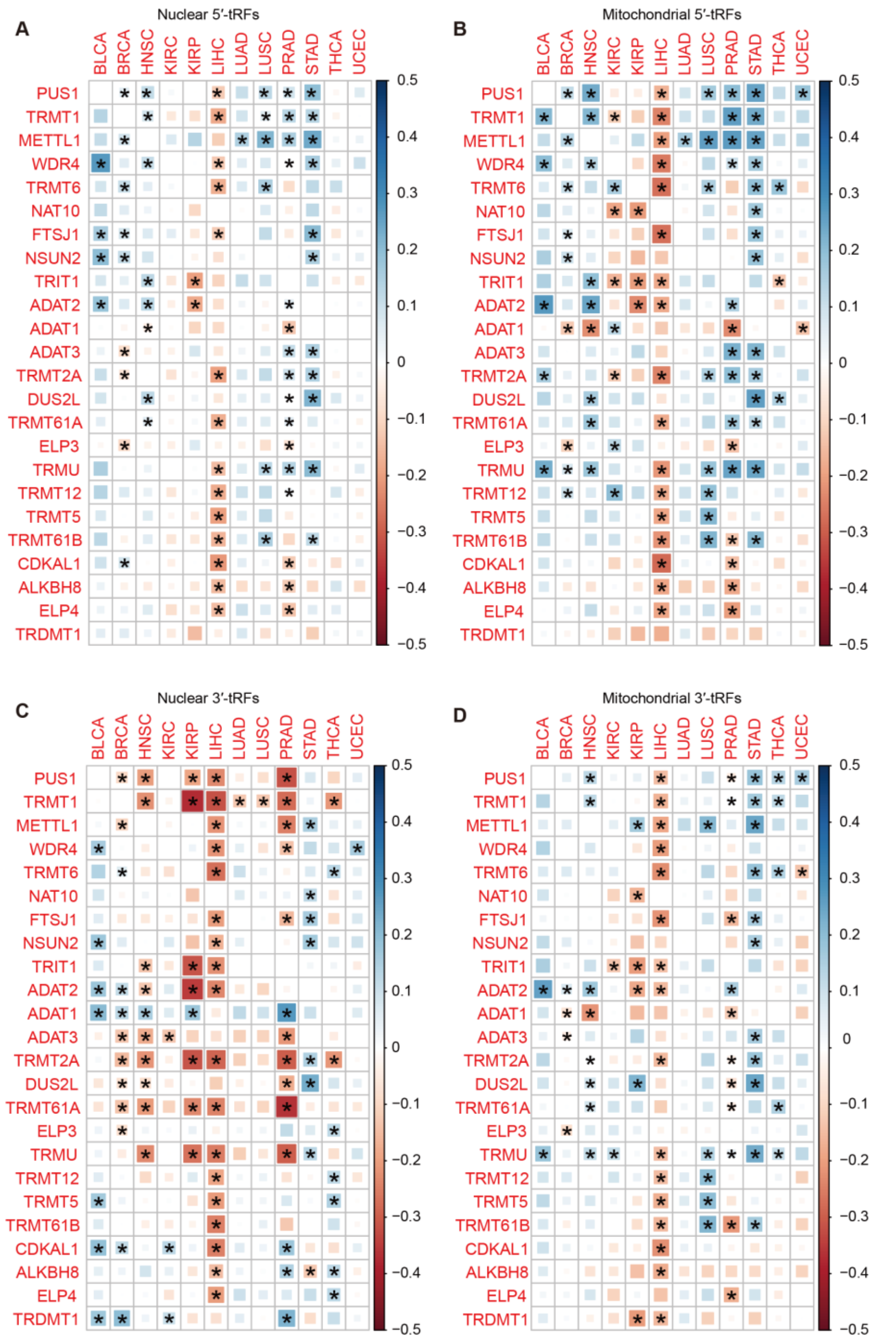

Supplementary Fig. S11. Association of tRNA modification enzymes with 5′-tRF from nucleus (A), mitochondria (B), 3′-tRF from nucleus (C), and mitochondria (D) across cancer types. To assess associations of tRNA modifiers with tRFs, we calculated the median correlation between each tRNA modification enzyme and the expression levels of nuclear and mitochondrial tRFs in each cancer type. We then evaluated the statistical significance of median correlations by permuting the expression of tRNA modifying enzyme 1,000 times. **P* <0.05 with permutation test.

Notes: Supplementary Table S1-S7 in the format of Excel files can be downloaded separately**.**

Supplementary Table S1, related to Fig. 1. Summary of TCGA samples used in this study and summary of 5′-tRFs, 3′-tRFs and 3′U-tRFs identified in this study.

Supplementary Table S2, related to Fig. 2. Summary of total reads mapped to 5′-tRFs, 3′-tRFs and 3′U-tRFs in our miRNA-seq data set of 18 lung tumors and matched normal tissues from non-small cell lung cancer patients.

Supplementary Table S3, related to Fig. 3. Summary of dysregulated 5′-tRFs and 3′-tRFs across different cancer types.

Supplementary Table S4, related to Fig. 4. (**A**) Clinical characteristic analyses of KIRC patients in TCGA. (**B**) Cox analyses of tRF expression subtypes and prognostic signatures adjusting for TNM stage or SSIGN score.

Supplementary Table S5, related to Fig. 5. Summary of 5'-tRF and 3′-tRF expression subtypes and subtype-specific signatures across cancer types.

Supplementary Table S6. related to Fig. 6. Tissue specificity of 11 cancer driver tRFs.

Supplementary Table S7, related to Fig. 7 and "Supplementary Methods". Information on antibody and primers used in this study.

Supplementary References

1. Li, H. and Durbin, R. (2009) Fast and accurate short read alignment with Burrows-Wheeler transform. *Bioinformatics*, **25**, 1754-1760.
2. Cabili, M.N., Trapnell, C., Goff, L., Koziol, M., Tazon-Vega, B., Regev, A. and Rinn, J.L. (2011) Integrative annotation of human large intergenic noncoding RNAs reveals global properties and specific subclasses. *Genes Dev*, **25**, 1915-1927.
3. Frank, I., Blute, M.L., Cheville, J.C., Lohse, C.M., Weaver, A.L. and Zincke, H. (2002) An outcome prediction model for patients with clear cell renal cell carcinoma treated with radical nephrectomy based on tumor stage, size, grade and necrosis: the SSIGN score. *The Journal of urology*, **168**, 2395-2400.
4. Kim, H. and Park, H. (2007) Sparse non-negative matrix factorizations via alternating non-negativity-constrained least squares for microarray data analysis. *Bioinformatics*, **23**, 1495-1502.
5. Barbie, D.A., Tamayo, P., Boehm, J.S., Kim, S.Y., Moody, S.E., Dunn, I.F., Schinzel, A.C., Sandy, P., Meylan, E. and Scholl, C. (2009) Systematic RNA interference reveals that oncogenic KRAS-driven cancers require TBK1. *Nature*, **462**, 108-112.
6. Akbani, R., Ng, P.K., Werner, H.M., Shahmoradgoli, M., Zhang, F., Ju, Z., Liu, W., Yang, J.Y., Yoshihara, K., Li, J. *et al.* (2014) A pan-cancer proteomic perspective on The Cancer Genome Atlas. *Nat Commun*, **5**, 3887.
7. Honda, S. and Kirino, Y. (2015) Dumbbell-PCR: a method to quantify specific small RNA variants with a single nucleotide resolution at terminal sequences. *Nucleic acids research*, **43**, e77.
8. Honda, S., Shigematsu, M., Morichika, K., Telonis, A.G. and Kirino, Y. (2015) Four-leaf clover qRT-PCR: A convenient method for selective quantification of mature tRNA. *RNA biology*, **12**, 501-508.
